# Artificial Neural Network (ANN) and Finite Element (FEM) Models for GFRP-Reinforced Concrete Columns under Axial Compression

**DOI:** 10.3390/ma14237172

**Published:** 2021-11-25

**Authors:** Haytham F. Isleem, Bassam A. Tayeh, Wesam Salah Alaloul, Muhammad Ali Musarat, Ali Raza

**Affiliations:** 1 Department of Civil Engineering, Tsinghua University, Beijing 180004, China; 2 Department of Civil Engineering, Faculty of Engineering, Islamic University of Gaza, Gaza P.O. Box 108, Palestine; btayeh@iugaza.edu.ps; 3 Department of Civil and Environmental Engineering, Universiti Teknologi PETRONAS, Bandar Seri Iskandar, Tronoh 32610, Malaysia; muhammad_19000316@utp.edu.my; 4 Department of Civil Engineering, University of Engineering and Technology, Taxila 47050, Pakistan; ali.raza@uettaxila.edu.pk

**Keywords:** glass fiber-reinforced polymer (GFRP), hollow concrete-core (HCC), axial load-axial strain, confinement of columns, ductility, hardening behavior

## Abstract

In reinforced concrete structures, the fiber-reinforced polymer (FRP) as reinforcing rebars have been widely used. The use of GFRP (glass fiber-reinforced polymer) bars to solve the steel reinforcement corrosion problem in various concrete structures is now well documented in many research studies. Hollow concrete-core columns (HCCs) are used to make a lightweight structure and reduce its cost. However, the use of FRP bars in HCCs has not yet gained an adequate level of confidence due to the lack of laboratory tests and standard design guidelines. Therefore, the present paper numerically and empirically explores the axial compressive behavior of GFRP-reinforced hollow concrete-core columns (HCCs). A total of 60 HCCs were simulated in the current version of Finite Element Analysis (FEA) ABAQUS. The reference finite element model (FEM) was built for a wide range of test variables of HCCs based on 17 specimens experimentally tested by the same group of researchers. All columns of 250 mm outer diameter, 0, 40, 45, 65, 90, 120 mm circular inner-hole diameter, and a height of 1000 mm were built and simulated. The effects of other parameters cover unconfined concrete strength from 21.2 to 44 MPa, the internal confinement (center to center spiral spacing = 50, 100, and 150 mm), and the amount of longitudinal GFRP bars (*ρ_v_* = 1.78–4.02%). The complex column response was defined by the concrete damaged plastic model (CDPM) and the behavior of the GFRP reinforcement was modeled as a linear-elastic behavior up to failure. The proposed FEM showed an excellent agreement with the tested load-strain responses. Based on the database obtained from the ABAQUS and the laboratory test, different empirical formulas and artificial neural network (ANN) models were further proposed for predicting the softening and hardening behavior of GFRP-RC HCCs.

## 1. Introduction

The use of glass fiber-reinforced polymer (GFRP) rebars as a replacement for the internal steel reinforcement is an excellent solution to have durable reinforced concrete (RC) structures in aggressive environments [1,2]. Therefore, many studies have concentrated on understanding and simulating the axial behavior of concrete members with internal FRP bars (i.e., [3,4,5,6,7,8,9,10,11,12,13,14]). Among them, hollow concrete-core columns (HCCs) are widely used in civil infrastructures including bridge piers, ground piles, and utility poles to minimize the total weight and reduce costs. HCCs are also considered in practice to increase the strength-to-mass ratio of structures compared with solid concrete columns [15,16,17,18]. Designing HCCs with adequate strength and ductility, however, requires careful consideration of important variables, such as the details of the hoop confinement and compressive strength of concrete [15,19,20,21]. HCCs tested in [15] with a larger spacing of hoop reinforcement result in brittle failure, premature buckling of vertical bars, and reduced deformation. Lee et al. [18] reported that decreasing the spacing of the lateral reinforcement from 80 mm to 40 mm enhanced the ductility by 20% and minimized the damage associated with the inner concrete core. In addition, Mo et al. [19] found that when the concrete compressive strength increased from 30 MPa to 50 MPa, it yielded stiffer resistance in compressed HCCs, but with up to a 50% reduction in deformation capacity because of faster crack propagation and concrete splitting. Based on these researches, the ductility capacity of HCCs is significantly influenced by hoop confinement details, and the failure mode depends largely on the grade of concrete compressive strength. 

Moreover, one of the major aspects influencing the seismic capacity of HCCs is the aspect ratio (length to cross-section depth). The failure pattern and the mechanical performance of the hollow concrete-core columns depend on the effect of the amount of longitudinal reinforcement [22,23]. Various researches examine the mechanical performance of HCCs with internal steel bars under different loading conditions [18,19,22,23,24,25,26,27,28]. These studies concluded that the structural behavior of HCCs depends on the ratio of inner to the outer diameter of the HCCs (*Di*/*D*), axial-loading ratio, amount of longitudinal reinforcement, and the amount of the lateral confinement. Furthermore, when the *D_i_*/*D* was increased while keeping the other variables constant, the ductility of steel-reinforced HCCs was decreased. Due to the brittle failure of HCCs under shear stresses, a sudden decrease in the axial compressive strength capacity occurred at the ultimate condition [29,30]. The reduced axial compressive strength was more prominent for the HCCs with larger inner-to-outer concrete-core diameter ratios [18,20]. Due to buckling failure and limited axial strains of steel, the axial performance of HCCs was not examined in the post-loading stage. Instead, when the steel bars start to buckle, the crushing of concrete material occurs [16,31,32]. 

On the other hand, increasing the inner-to-outer concrete-core diameter ratio in the GFRP-RC HCCs changed the columns’ failure pattern from brittle to ductile as reported by AlAjarmeh et al. [6]. After spalling of concrete cover, the failure in the HCCs with inner-to-outer concrete-core diameter ratio of 0.16 or 0.26 was initiated by the vertical and lateral GFRP-reinforcing rebars, while the failure of the HCCs with inner-to-outer concrete-core diameter ratio of 0.36 was initiated by crushing of the hollow concrete part. AlAjarmeh et al. [6] found that the GFRP HCCs performed better than steel-reinforced HCCs with a higher inner-to-outer diameter ratio because of the higher contribution of GFRP bars to the overall stiffness of the column. The GFRP HCCs exhibited 11% higher axial capacity than the steel-reinforced HCCs. The GFRP-RC HCCs showed 22% and 54% higher ductility and confinement efficiency than the steel RC HCCs, respectively. In addition, AlAjarmeh et al. [5] found that the increase in the diameter and number of longitudinal GFRP bars increased the strength and ductility of the GFRP HCCs. 

Many analytical models for predicting the strength and ductility of steel-confined concrete are now available in the literature [33,34,35]. However, designing concrete structures with GFRP bars is not straightforward by using steel-reinforced concrete design formulas and guidelines due to the different properties of FRP bars compared to steel. This is because concrete columns confined by steel hoops behave differently from columns confined by internal FRP hoops, and if these models are applied to columns with internal FRP reinforcement, the strength, strain, and structural response may be inaccurately estimated. One of the challenges that often face engineers is to estimate the maximum compressive strength and ductility capacities of FRP-reinforced concrete columns accurately. This can be achieved using destructive methods through laboratory tests or non-destructive methods such as analytical design models. A monotonic stress-strain model as an envelope response of the cyclic stress-strain model is also necessary for the seismic analysis of FRP-confined RC columns based on fiber approach [36] or finite element model [37,38]. 

Therefore, many researchers worked on the finite element modeling (FEM) of GFRP or CFRP confined solid concrete columns under different loading conditions [39,40,41,42,43,44,45,46]. These researches confirmed that the FEM simulation can capture the failure pattern and the structural performance of FRP-RC columns accurately. The FEM explicitly deals with all the deficiencies of the mathematical models. The FE simulations can save time and cost as compared with tests by creating suitable computational models that can simulate the complex damaging behavior of FRP composites accurately [47]. To simplify the FEM and to speed up the FE simulations, it is essential to make some assumptions, but it is also important to implement the actual experimental testing environments in FEM. It is suggested to overcome the model’s complexities by choosing the accurate types and sizes of elements to significantly reduce the simulation period and to improve the performance of the numerical findings. Therefore, as stated in Ref. [48] the FEM using ABAQUS software [49] with strong background knowledge is the most efficient tool to solve various engineering problems. 

The recent work conducted in [50] consists of two aims: the first aim is to propose a new FEM model for predicting the axial structural performance of HCCs reinforced with GFRP bars, and the second aim is to propose the new empirical models for predicting the first and second peak loads of HCCs reinforced with GFRP rebar. To achieve the major goal of the present paper, a finite element model for HCCs reinforced with GFRP bars was constructed using the ABAQUS software [49]. This model was calibrated for various material and geometric parameters of the test specimens using experimental results from [5]. To date, all the available models regarding FRP-RC hollow columns have not yet, however, considered the effect of several test variables as discussed previously such as lateral spiral spacing, concrete compressive strength, and the ratio of inner to the outer diameter of the HCCs on the failure mode, load-displacement behavior, ductility, and strength of hollow concrete-core columns.

In addition, authors are now using the power of advanced learning methods in civil infrastructure systems, and many studies that successfully estimate the mechanical performance of steel- or FRP-confined concrete have been published (i.e., [51,52,53,54,55,56]). Among the studies, Oreta and Kawashima [51] have used artificial neural networks (ANN) to estimate the axial strength and strain of compressed steel-confined concrete columns of circular sections. An ANN model with input variables including the concrete‘s compressive strength, the concrete-core diameter, the column height, the longitudinal reinforcement ratio, and the effectiveness of hoop confinement was reported. The ANN models are shown to be very important in simulating physical processes. The ANN models are also found to provide better results compared with some Regression Analysis (RA) models.

The present paper aims to provide a full stress-strain model based on the available experiments and findings of GFRP-RC HCCs. The model consists of different approaches of ANN modeling and regression-based models. The database used in the analysis contains the experimental results of axial compression tests on 17 circular specimens reinforced with internal longitudinal GFRP rebars and spirals. It is to be noted that the available studies on GFRP HCCs [5,6,7] have considered a limited range of inner-to-outer core diameter ratios. These diameters were chosen based on commercially available PVC pipes and resulted in inner-to-outer concrete-core diameter ratios similar to those studied in [16,32,57,58]. This model was, therefore, calibrated for various material and geometric parameters of specimens using the load-strain results in [5,6,7] as well as new test specimens constructed with the help of ABAQUS software [49]. Due to limitations in the column tests, a full database of 60 FRP-reinforced concrete members was obtained using Finite Element Analysis (FEA) ABAQUS to secure several objectives of the present paper. An easy-to-use approach is then provided for predicting the compressive strength and strain of FRP-RC columns. The proposed models for the design of FRP-confined RC columns can guarantee a safe design in regards to new column parameters.

## 2. Research Significance 

The design of FRP-reinforced concrete members under flexural loading has been documented in the literature [59,60,61]. However, it is recommended by these guidelines to ignore the axial capacity contributed by the longitudinal FRP bars in the section under compression. The use of FRP rebars as reinforcement in concrete members under compression is not suggested by the ACI code [59] due to uncertainty and inadequate information regarding the variation in properties of compressed FRP bars. CSA-S806-12 [60] suggests using the FRP bars in compression members without taking into account the compression contribution of FRP bars for designing. The reinforcing GFRP bars in concrete columns were investigated in several experimental studies in the last period (i.e., [60,62,63,64,65,66,67,68,69]), which led to the introduction of many theoretical and numerical models. Table 1 shows a summary of these models. The existing tests and proposed models confirmed that when the axial capacity of the longitudinal FRP rebars is ignored, the overall column capacity is underestimated [3,70]. According to Tobbi et al.’s [3] study on GFRP-RC columns, the contribution made by the longitudinal FRP rebars at the peak loading condition was 35% of their maximum strength in tension. Elmessalami et al. [71] have recently reviewed and analyzed the tests available on FRP-reinforced concrete columns and found that the GFRP rebars provide an enhancement of about 3 to 14% at peak load and that CFRP bars give 6 to 19% mainly based on the FRP reinforcement ratio. Considering these findings and determining the axial compression capacity of the column by taking the axial strain of the FRP rebars equal to that of the unconfined concrete at peak load (i.e., 0.003) reveals excellent agreement with the test findings [11]. 

However, it is to be noted that all of these experimental tests and theoretical investigations have been focused largely on solid FRP-reinforced concrete members. Furthermore, the proposed models mainly estimate the maximum peak axial capacity in the elastic region of the axial load-axial strain response. Compared with GFRP-reinforced solid concrete columns, the hollow columns with internal longitudinal and lateral GFRP reinforcement exhibited significantly different failure patterns and structural responses [6]. Due to the reduced effective concrete area, the hollow columns failed at a reduced axial load compared with that of the solid columns. In this paper, to gain sufficient knowledge on the effect of the published models on stress predictions, Table 1 provides clear comparisons between the estimated errors (AAE, MSE, SD) obtained by comparing the theoretical predictions with the results of 60 HCCs provided in the present paper.

Generally, the comparisons of Table 1 reveal that all models that consider the strain in FRP bars to be similar to the peak strain of unconfined concrete (i.e., 0.003) (i.e., [63]), provide lower AAE errors as compared with the other models. However, an inspection of the comparisons with the strengths at the columns’ failures indicates that the AAE errors of the models are almost three times higher than those calculated at the peak load. If the results of these models are incorporated into an axial stress-strain model, the overall structural response of the columns may be significantly different than the experimental one. In light of research demands, the goal of the following sections of the present paper is to develop a new axial compression model that can accurately predict the axial load-axial strain response of concrete columns of both solid and hollow cross-sections reinforced with longitudinal GFRP rebars and spirals. 

## 3. Experimental Program

### 3.1. Tests Addressing the Effect of Longitudinal Reinforcement Ratio

**Figure 1:**
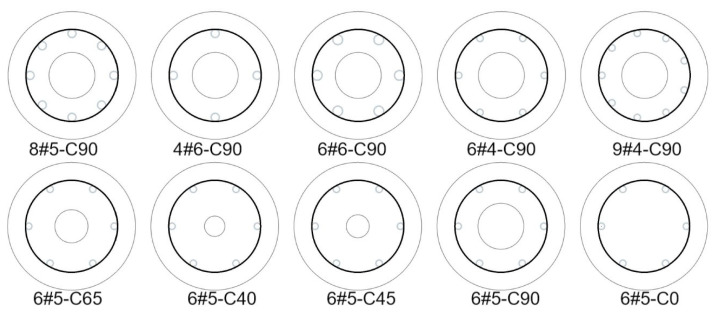
Cross-sectional and GFRP reinforcement details.

The tests in Ref. [5] aimed at addressing the effect of reinforcement ratio as the main design and test parameter on the behavior of hollow concrete-core columns. To achieve this goal, six GFRP-reinforced concrete columns 250 mm in diameter and 1000 mm in height were cast and tested. All the tested columns were reinforced longitudinally with sand-coated and high-modulus GFRP bars of different diameters (12.7, 15.9, or 19.1 mm) and amounts of longitudinal reinforcement but with the same configuration of lateral GFRP spirals. The GFRP spirals were spaced at 100 mm center-to-center in the vertical direction along 500 mm at the column mid-height and to avoid a sudden premature failure caused by the stress concentration spiral spacing of 50 mm within the rest of the column’s region is chosen. The inner-to-outer diameter ratio was constant at 0.36. Figure 1 shows the cross-sections and reinforcement details of the tested columns, and Table 2 provides the details of column geometry, configuration and amount of GFRP reinforcement, and material properties. The specimens corresponding to their tests in Table 1 were designated with the C that indicates the grade of concrete (i.e., C25), followed by the spiral spacing (i.e., H100) and then the number and size of GFRP vertical reinforcement. The last code refers to the inner-hole diameter. For example, specimen C25-H100-6#4-90 is a hollow concrete-core column reinforced with six #4 GFRP bars and the diameter of the inner hole is 90 mm. All columns in these tests were made with ready-mix concrete and the average concrete compressive strength at 28 days was 25.0 MPa. 

### 3.2. Tests Addressing the Effect of Inner Void’s Size 

The goal of the tests in Ref. [6] is to evaluate the effect of the inner-to-outer concrete-core diameter ratio on the structural response and failure model of both GFRP and steel-reinforced hollow concrete-core columns. To achieve this goal, five concrete columns 250 mm in diameter and 1000 mm in height were cast and tested. Among them, four columns were constructed with GFRP bars and spirals, while only one column was constructed with longitudinal steel bars and GFRP spirals. It is to be noted that the present paper has not considered modeling steel-reinforced concrete columns. All the tested five columns were reinforced with six longitudinal rebars and GFRP spirals with a center-to-center spacing of 100 mm along 500 mm length at the column mid-height, and to avoid premature failure by stress concentration a vertical spacing of 50 mm within 250 mm in the other 250 mm regions of the columns was chosen. A total of 6 longitudinal GFRP and steel bars were used comprising the same reinforcement ratio of 2.79%, which is within the recommended range of 1–4%. To determine the effect of the inner hollow core size. The details of the reinforcement were kept similar for all tested columns. The inner-hole diameters were 40 mm, 65 mm, and 90 mm. A solid concrete column was also prepared and tested as a reference specimen. A concrete column with a maximum diameter of 250 mm was considered due to the limited capacity of the test equipment. A diameter of 90 mm for the inner core was selected for a sufficient concrete cover for the longitudinal rebars. Similarly, a hollow column with an inner diameter of 65 mm (*D_i_*/*D* ratio = 0.26) and reinforced with the same reinforcement details with six 16 mm steel bars was taken as a benchmark for comparison with the GFRP-reinforced columns. Figure 1 shows the cross-sections and reinforcement details of the tested columns, and Table 2 provides the full details of the specimens and reinforcement materials. Similarly, the specimens corresponding to their tests in Table 1 were designated with the C that indicates the grade of concrete (i.e., C31.8), followed by the spiral spacing (i.e., H100) and then the amount of vertical GFRP-reinforcing bars and their size. The last code refers to the inner-hole diameter. For example, specimen C31.8-H100-6#5-65 is a hollow concrete-core column reinforced with six #5 GFRP bars and the diameter of the inner hole is 65 mm. The average concrete compressive strength for all specimens at 28 days was around 31.8 MPa.

### 3.3. Tests Addressing the Effect of Varying Amount of Internal GFRP Confinement

The tests in Ref. [7] aimed at investigating the effectiveness of GFRP bars and spirals as internal reinforcement in HCCs. It focused on evaluating the effect of lateral spiral spacing and concrete compressive strength on the failure mode, load-displacement behavior, ductility, and confined strength of hollow concrete-core columns. To achieve these goals, seven concrete columns fully reinforced with GFRP bars with overall dimensions of 250 mm in diameter and 1000 mm in height were cast and tested. The column section and its maximum capacity were carefully determined to be successfully tested by the machine. All columns were longitudinally reinforced with six GFRP bars following the reinforcement details, and ratio recommended in the AS3600 code [58] for steel reinforcement. Consequently, the reinforcement ratio of 2.79% was similar for all test columns. The inner-to-outer diameter ratio was constant at 0.36. Among all tests, three columns were reinforced laterally with GFRP spirals with spacings of 50, 100, and 150 mm at the middle portion of the samples (500 mm). Another column without lateral reinforcement at the testing region (500 mm) was prepared to evaluate the effect of the lateral reinforcement. These lengths were chosen to ensure crushing failure in the bars with lengths of 50, 100, and 150 mm, and bar buckling failure in the last sample. The remaining specimens were cast with different concrete strengths (21.2, 26.8, 36.8, and 44.0 MPa) and tested. These levels of compressive strength were considered normal-strength concrete, as indicated in the ACI 318 code [59]. The details of the reinforcing bars for all tested columns were similar, in which the longitudinal reinforcement ratio was 2.79% and 100 mm spacing between lateral spirals was chosen to determine the effect of varying compressive strengths of unconfined concrete. Figure 1 shows the cross-sections and reinforcement details of the tested columns, and Table 2 provides the full details of the specimens and reinforcement materials. Similarly, the specimens corresponding to their tests in Table 1 were designated with the C that indicates the grade of concrete (i.e., C21.2, 26.8, 36.8, 44.0 MPa), followed by the spiral spacing (i.e., H100), and then the amount of GFRP rebars in the longitudinal direction and their size. The last code refers to the inner-hole diameter. For example, specimen C44.0-H100-6#5-90 is a hollow concrete-core column reinforced with six #5 GFRP bars and the diameter of the inner hole is 90 mm.

### 3.4. Tests with the Help of ABAQUS Software

The Finite Element (FE) experiments consist of 43 specimens with similar dimensions of 250 mm in diameter and 1000 mm in height. These specimens considered a new range of test parameters. An example is that Alajarmeh et al. [6] have focused on assessing the influence of the inner-to-outer diameter ratio on the structural performance of HCCs, while all their columns were reinforced and constructed with the same materials and reinforcement configurations. The present paper, therefore, expanded the range of investigated *D_i_*/*D* ratio from 0.16 to 0.48 (i.e., C44.0-H100-6#5-120, C31.8-H100-6#5-120, C26.8-H50-6#5-120, C26.8-H100-6#5-120, C26.8-H150-6#5-120, C21.2-H100-6#5-120) to overcome the limited sizes of PVC pipes that are available in the market. For a comprehensive development of an empirical axial stress-strain model, solid concrete columns were also provided (C31.8-H100-6#5-00, C26.8-H50-6#5-00, C26.8-H150-6#5-00, C21.2-H100-6#5-00, C44.0-H100-6#5-00, C26.8-H100-6#5-00). The detail of the columns’ cross-sections and the configuration and amount of the GFRP reinforcing rebars. 

## 4. Finite Element Modeling

### 4.1. Model Geometry, Interaction, Loading, and Boundary Conditions

This sub-section provides the details of the finite element models for predicting the structural response of GFRP-reinforced HCCs tested under axial compression loads. The numerical simulations of HCCs were performed using the commercial ABAQUS software [49]. The initial stiffness, crack propagation behavior, peak load, post-peak stiffness, and failure mechanism of HCCs were considered by the proposed FEM model. The complexity in simulating the damage behavior of confined concrete was done by using a proposed damaged plastic model, and the behavior of GFRP rebars was modeled as linear elastic material up to failure. To check the performance of the proposed FEM model, the experimental results of 17 HCCs were compiled from [5,6,7].

**Figure 2:**
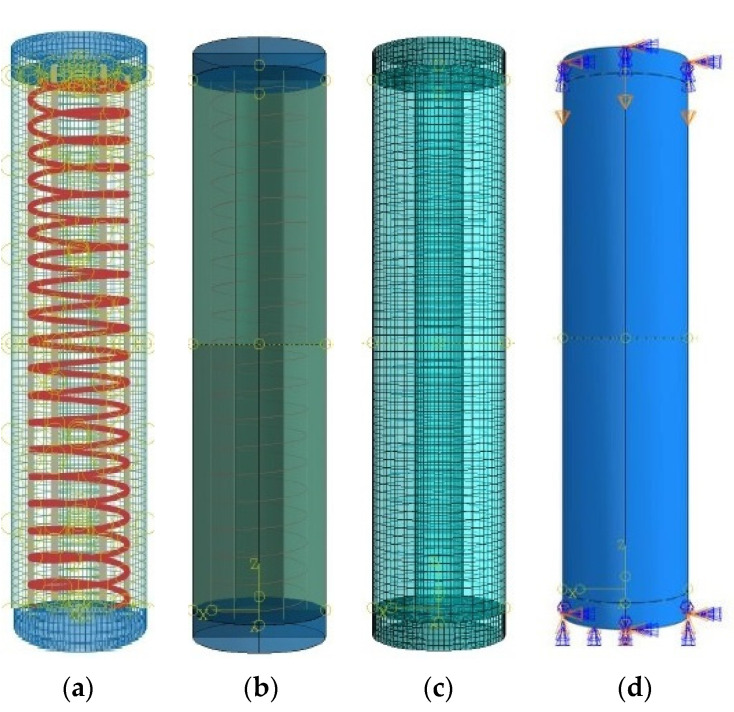
Simulated model of HCCs showing GFRP reinforcement (**a**); Material sections (**b**); Element meshing (**c**), and Boundary condition (**d**). To be able to describe relationships between the elements of the structures, the figure also shows a three-dimensional coordinate axis system.

The concrete and steel plates were modeled as homogenous 3-dimensional solid stress sections and were assigned C3D8R element types. The GFRP rebars were modeled as 3-dimensional deformable wire elements and were assigned T3D2 element types. The HCCs were fixed at the bottom and top ends, and only free to translate in the vertical direction at the top end (*θx*, *θy*, *θz* = 0; *Ux* and *Uy* = 0, *Uz* ≠ 0). The interaction between the concrete material and the GFRP-reinforcing rebars was defined using the ‘embedded region’ constraint provided by ABAQUS software [49] that connects the compatible degrees of freedom (DOF) of the truss elements of reinforcement bars to the required DOF of the 3-dimensional stress elements of concrete [71]. Using the displacement control method, a value of 50 mm was applied on the top end plate for all the specimens. The steel plates of 50 mm thickness to apply the boundary conditions and transfer the loads on column cross-section were connected using ‘tie’ constraint on the top and bottom ends of the columns for the application of the boundary conditions. While applying the ‘tie’ constraint between the steel plates and column, the bottom surface of the top steel plate was considered as a master surface and the top surface of the HCCs was considered as a slave surface. Similarly, the bottom surface of HCCs was taken as a slave surface and the top surface of the bottom steel plate was taken as a master surface. The geometry and modeling details of the simulated GFRP-reinforced concrete specimens with the applied boundary conditions are shown in Figure 2, whereas the key characteristics of the FE model are summarized in Table 3, in which the mesh size of 10 mm for the reinforcement bars provides good performance. Due to the earlier fracture of the longitudinal reinforcement and their insignificant effects on the column performance, the mesh size regarding different bar sizes shows no major impact. More tests with different spiral sizes, which possess a significant effect during the simulation period and, overall, the column response, should be investigated. 

### 4.2. Material Simulations

#### 4.2.1. Concrete

The simulation of the behavior of concrete material is one of the challenging tasks due to its complex nature. The behavior of concrete was, in the reversible regime, defined by Young’s modulus and the Poisson’s ratio. The Young’s modulus value was provided by Equation (1) [72] and the Poisson’s ratio value was 0.2 [73]. The density of the normal weight concrete was commonly taken as 2400 kg/m^3^. For the definition of the irreversible regime of concrete, various models are available in ABAQUS such as the concrete damaged plastic model (CDPM), brittle crack model (BCM), smeared cracking model (SCM), and Drucker–Prager model (DPM). In the current work, CDPM was utilized to simulate the inelastic nature of concrete. This model can capture the complex nature of concrete material by considering the cracking and crushing of concrete. Therefore, it is commonly accepted while simulating the nonlinearity of concrete [74,75,76]. The CDPM considers the definition of various properties of concrete such as tensile behavior, compressive behavior, plastic behavior, and damaging behavior of concrete:

(1) $$E_{c}=3320\sqrt{f_{c}^{’}}+6900$$

where *f_c_^’^* is the compressive stress of concrete material tested at 28 days. 

**Figure 3:**
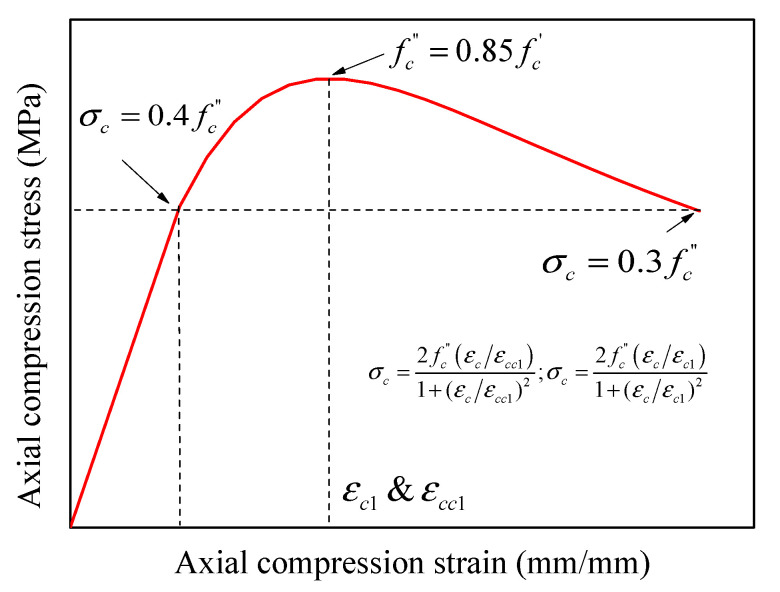
Stress-strain models for unconfined and confined concrete.

The plastic behavior of concrete as defined by *ABAQUS User Manual 6.14* [49], considers five parameters of concrete as follows: the dilation angle (*ψ*), the viscosity parameter, the eccentricity (*ε*), the ratio of biaxial to uniaxial stresses (*σ_bo_*/*σ_co_*) [65], and the shape factor of yielding surface (*Kc*) (see Table 4). All these factors were calibrated to obtain the best results as compared with the test measurements. To calculate uniaxial compressive stresses of concrete (*σ_c_*), Equation (2) was proposed in the current research, mainly based on models provided in the literature (i.e., [77]) but with some modifications to account for unconfined and confined concrete as reported by Zeng et al. [78] (i.e., Equation (4)). For the definition of compressive behavior of concrete, the compressive stiffening model (Equation (2)) is presented in Figure 3 for a selected specimen.

(2) $$\sigma_{c}=\left\{ \begin{array}{l} \frac{2f_{c}^{’’}\left(\varepsilon_{c}/\varepsilon_{cc1}\right)}{1+{\left(\varepsilon_{c}/\varepsilon_{cc1}\right)}^{2}};\\ \frac{2f_{c}^{’’}\left(\varepsilon_{c}/\varepsilon_{c1}\right)}{1+{\left(\varepsilon_{c}/\varepsilon_{c1}\right)}^{2}} \end{array}\right.$$

where *f_c_^’’^*= 0.85 *f_c_^’^* as considered in the [79] model. Respectively, the terms *ε_c_*_1_ and *ε_cc_*_1_ are the peak strains of unconfined and confined concrete (mm/mm), which are calculated using:

(3) $$\varepsilon_{c1}=0.0014\left[2-e^{-0.024f_{c}^{’’}}-e^{-0.140f_{c}^{’’}}\right]$$

(4) $$\varepsilon_{cc1}=\varepsilon_{c1}+800{\left(I_{e}\right)}^{0.2}\times 10^{-6}$$

(5) $$I_{e}=\frac{\rho_{v}f_{FRP}}{f_{c}^{’}}$$

where *I_e_* is the effective confinement index (non-dimensionless parameter); *ρ_v_* is the volumetric ratio of GFRP spirals.

Similarly, the strains model (Equations (3) and (6)) proposed by Majewski [80] were used in the present research for the predictions of strains of GFRP-RC HCCs. The strain provided in Equation (6) was used to predict the ultimate strain of HCCs with no lateral confinement (spirals’ spacing = 500 mm in the present tests). It was assumed in the present model that the compressive concrete strength in these two expressions to be *f_c_^’’^*. The uniaxial compressive strength of unconfined concrete cylinders (100 mm × 200 mm as tested in [5,6,7]) is usually higher than that of standard cylinders (150 mm × 300 mm); therefore, the strength was multiplied by a conversion factor of 0.85 to consider column size effects in the models proposed based on standard cylinders:

(6) $$\varepsilon_{cu}=0.004-0.0011\left[1-e^{-0.0215f_{c}^{’’}}\right]$$

**Figure 4:**
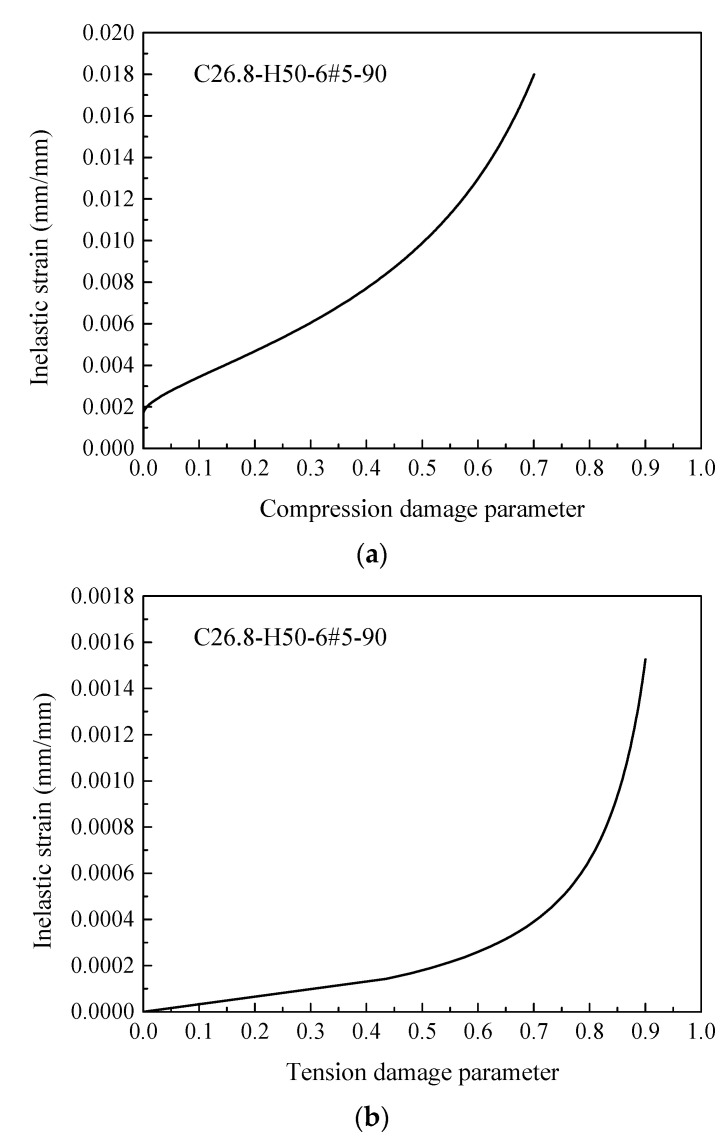
Relationship between the damage parameter and the inelastic strain of concrete for specimen C26.8-H50-6#5-90: (**a**) Represents the compression behavior; (**b**) Represents the tension behavior.

The elastic portion of the stress-strain curve can be taken up to 40% of the ultimate compressive strength of concrete, and the stress in the post-peak branch was considered to be 30% of the ultimate compressive strength of concrete (i.e., [81,82]) (see Figure 3). The uniaxial compression and tension damage parameters of concrete in the CDP model were defined using Equations (7) and (8), respectively. Considering a selected specimen from Table 2, Figure 4a,b present the predicted relationships between these parameters and the inelastic strains:

(7) $$d_{c}=1-\left(\frac{\sigma_{c}}{f_{c}^{’’}}\right)$$

(8) $$d_{t}=1-\left(\frac{\sigma_{t}}{f_{t}}\right)$$

where the maximum tensile strength of concrete (*f_t_*) was estimated using Equation (9), which was proposed in the present paper based on test data published in [83]:

(9) $$f_{t}=\left[11.954\exp\left(-0.007*f_{c}^{’’}\right)/100\right]f_{c}^{’’}$$

**Figure 5:**
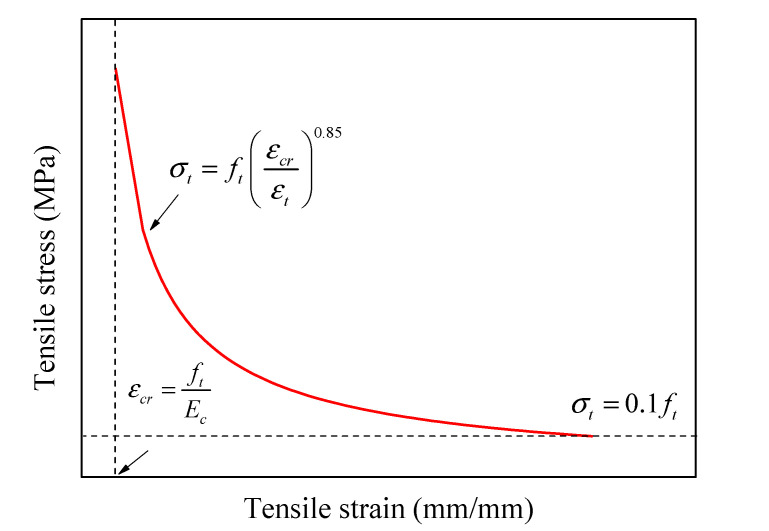
Model for the definition of tension stiffening of concrete.

The cracking strain (*ε_cr_*) corresponding to *f_t_* was determined as *ε_cr_* = *f_t_*/*E_c_*. The compression and tension strain of concrete in the inelastic region are respectively calculated by:

(10) $$\varepsilon_{c}^{in}=\varepsilon_{c}-\varepsilon_{oc}^{el}$$

(11) $$\varepsilon_{t}^{in}=\varepsilon_{t}-\varepsilon_{ot}^{el}$$

where *ε_oc_^el^* and *ε_ot_^el^* are the elastic strains of concrete under compression and tension stresses and calculated respectively as *ε_oc_^el^* = *σ_c_*/*E_c_* and *ε_ot_^el^* = *σ_t_*/*E_c_*. Figure 5 finally defines the behavior of unconfined concrete under tensile loads (Equation (12)) [84]:

(12) $$\sigma_{t}=f_{t}{\left(\frac{\varepsilon_{cr}}{\varepsilon_{t}}\right)}^{0.85}$$

**Figure 6:**
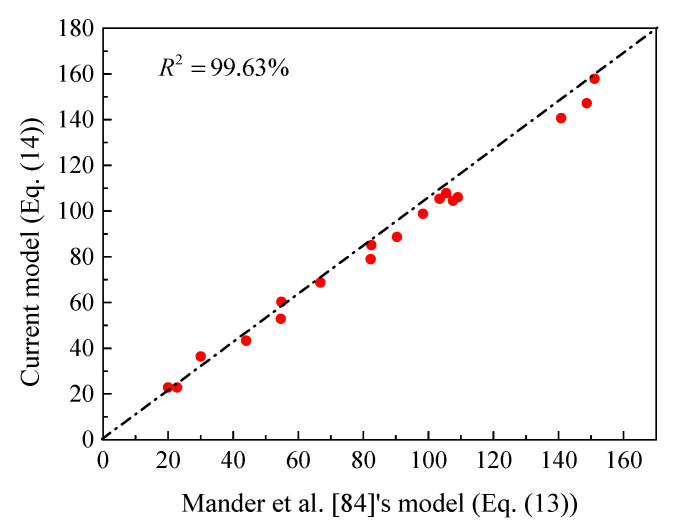
Correlation between the present confined strength model and that proposed by Mander et al. [92] for confined concrete.

The confinement made by the FRP spirals provides enhancements in the compressive strength and deformation of concrete [85]. This enhancement is usually higher at ultimate than that at peak condition resulting in a hardening stress-strain behavior for sufficiently confined concrete [86,87,88,89,90,91,92,93,94,95,96,97,98,99,100,101]. As the final failure was due to ruptures in the longitudinal GFRP bars and crushing of the concrete core at mid-height without damage to the lateral spirals [5,6,7], the confinement strength model proposed by Mander et al. [92] (represented by Equation (13)) was, therefore, used to exactly capture the compressive strength of confined concrete core (*f_cc_^’^*) obtained from the FE simulations. In case of exceeding the range of test parameters that were used to build Mander et al.’s [92] model, Equation (14) was herein introduced to have a different form. Finally, an excellent calibration between these two expressions is revealed by Figure 6:

(13) $$f_{cc}^{’}=f_{c}^{’’}\left(2.254\sqrt{1+\frac{7.94f_{l}}{f_{c}^{’’}}}-2\frac{f_{l}}{f_{c}^{’’}}-1.254\right)$$

(14) $$f_{cc}^{’}=f_{c}^{’’}+\left[191.17{\left(\frac{f_{l}}{f_{c}^{’’}}\right)}^{-0.67}{\left(1+\frac{D_{i}}{D_{s}}\right)}^{0.71}-8.52\left(\frac{f_{l}}{f_{c}^{’’}}\right)\right]{\left(1+\frac{D_{i}}{D_{s}}\right)}^{0.75}$$

where *f_l_* (MPa) is the confinement stress due to FRP spirals that can be calculated using the following well-known expression used for confined concrete externally reinforced with FRP wraps (i.e., [86,87,88,89,90,91,92,93,94,95,96,97,98,99,100,101]). To account for the effectively confined concrete core of spirally reinforced HCCs as in this present paper, some modifications were also introduced in Equation (15) to use the proposed next form:

(15) $$f_{l}=\frac{2E_{FRP}\varepsilon_{h,rup}t_{FRP}}{D}$$

(16) $$f_{l}=\frac{2f_{bent}d_{h}}{D_{s}-D_{i}}$$

where, in Equation (15), *E_FRP_* (MPa) = tensile elasticity modulus of FRP wraps; *t_FRP_* (mm) = thickness of all FRP wrapping layers; in Equation (16), the modifications are represented as follows: The total fiber sheets (*t_FRP_*) was replaced by the spiral diameter dh (mm). The diameter of a circular section wrapped with FRP (*D*) was replaced with the diameter of the effectively confined concrete core (*D_s_* − *D_i_*) (mm). Finally, the *E_FRP_* × *ε_h_*,*_rup_* (the tensile strength of the FRP wraps) was considered to account for the significantly reduced tensile capacity of the GFRP spirals at the bent portion when the straight FRP bar is bent to form the hoop reinforcement (i.e., [102,103,104,105]). In the present FEM model, the bent strength is found by Equation (17) as done by other researchers (i.e., [88]):

(17) $$f_{bent}=0.5f_{FRP}$$

**Figure 7:**
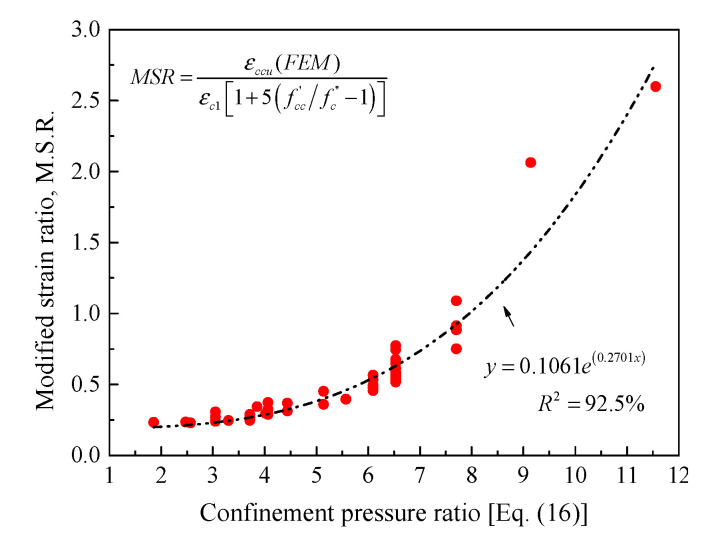
Correlation between the present confined strain model and that proposed by Mander et al. [92] for confined concrete.

Similarly, the strain of confined concrete at ultimate can be determined using Mander et al.’s [92] model (Equation (18)). It is to be noted that the unconfined concrete strain of 0.002 in their model was taken in the current model to be equal to the predicted εc1 value (Equation (3)). Moreover, to account for the effective confined concrete core of spirally reinforced HCCs as in the present paper, Equation (19) was suggested, based on the test results of all 60 specimens provided in Table 2. The test results of the 17 specimens tested in [5,6,7] were taken as a major control in the model development. An excellent correlation was finally obtained between the existing and currently proposed expressions (see Figure 7):

(18) $$\varepsilon_{ccu}=\varepsilon_{c1}\left[1+5\left(f_{cc}^{’}/f_{c}^{’’}-1\right)\right]$$

(19) $$\varepsilon_{ccu}=\varepsilon_{c1}\left[1+5\left(f_{cc}^{’}/f_{c}^{’’}-1\right)\right]\times M.S.R.$$

(20) $$M.S.R.=0.1061e^{\left(0.2701\times \frac{2f_{bent}d_{h}}{\left(D_{s}-D_{i}\right)f_{c}^{’’}}\right)}$$

#### 4.2.2. GFRP Bars

The GFRP reinforcement (longitudinal rebars and spirals) were considered as 3D deformable wires. An isotropic linear elastic material up to failure model was considered for simulating the behavior of the GFRP reinforcement [41,42,71]. The mechanical properties of GFRP bars used for the FEM were presented in Table 2 [5,6,7]. The behavior of FRP bars and ties was defined in terms of density (2.1 × 10^−9^ ton/mm^3^), elastic tensile modulus, ultimate strength, and Poisson’s ratio (0.25) [41]. The contribution of the longitudinal GFRP bars in compression was considered to be 50% of their maximum strength as straight bars having the same elastic modulus [2]. The contact between the GFRP-reinforcing rebars and the surrounding concrete was defined using the ‘embedded region’ constraint. 

#### 4.2.3. Steel End Plates

**Figure 8:**
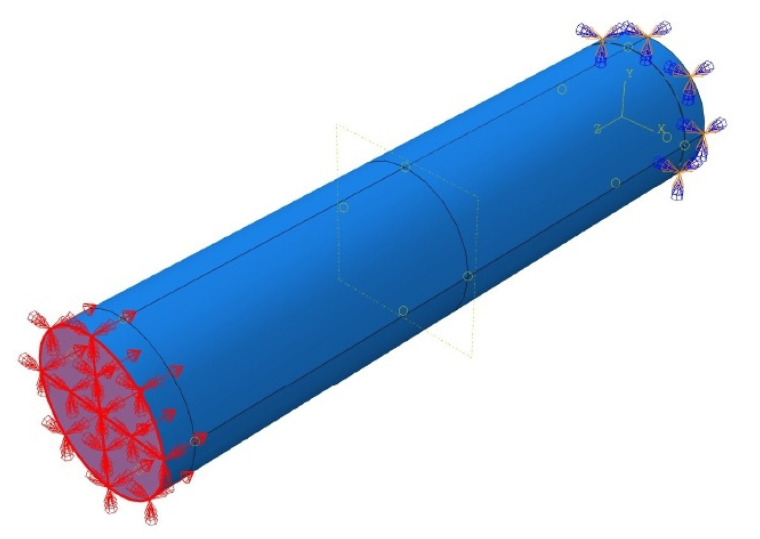
Proposed boundary conditions for GFRP-RC HCCs tested in the current paper.

The column specimens in the present simulations were tested with deformable 3D steel end plates. The boundary conditions chosen for these end plates are clearly shown in Figure 8. The material was considered steel with linear elastic behavior. The linear elastic behavior of the steel plates was, in the present paper, defined in terms of density (7.8 × 10^−9^ ton/mm^3^), elastic tensile modulus (*E_s_* = 200,000 MPa without any yield limit), and Poisson’s ratio of 0.30. 

### 4.3. FEM Results and Discussions

#### 4.3.1. Failure Mode

**Figure 9:**
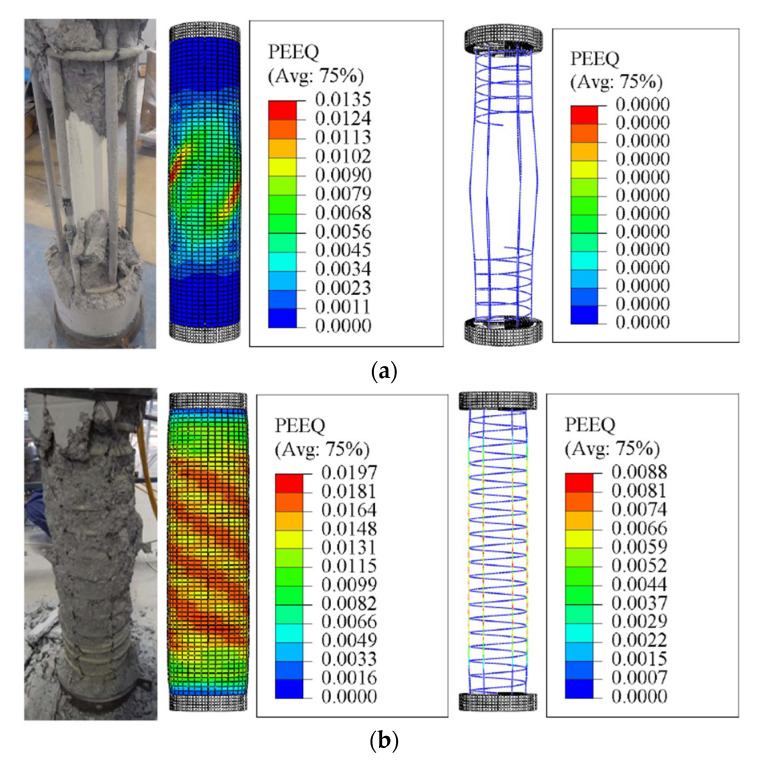
Experimental and finite element (FE) failure patterns of specimens (**a**) C26.8-H00-6#5-90; (**b**) C26.8-H50-6#5-90: Considering the effect of varying amounts of GFRP confinement. Note: PEEQ = Equivalent plastic strain.

According to the tests in [7], the failure in all columns started as vertically spreading hairline cracks appearing on the outer concrete surface at advanced loading levels. Once they appeared, the cracks propagated and widened, leading to different spalling features of the outer concrete cover, rupturing longitudinal GFRP bars, and damaging the concrete core. The different features of the column’s failure were mainly dependent on the effect of the column parameters. For example, specimen C26.8-H00-6#5-90 without internal GFRP confinement experimentally experienced explosive spalling and failing of both the concrete cover and core, causing large concrete pieces to fall from the specimen at column’s mid-height. Figure 9a reveals close agreement between the failed region and the global buckling of the longitudinal GFRP bars that obtained from the FEM as clearly indicated by the zero PEEQ value recorded at column’s mid-height: PEEQ values (the material’s inelastic deformation) are corresponding to the FEM failure of concrete. On the other hand, for confined specimen, gradual overall concrete-cover spalling was observed, followed by lateral expansion in the concrete core, which was confined by the GFRP spirals. The rupture of the longitudinal GFRP bars in different locations throughout the column’s height then occurs, i.e., C26.8-H50-6#5-90 in Figure 9b, in which the PEEG values are higher compared with the unconfined specimen. 

**Figure 10:**
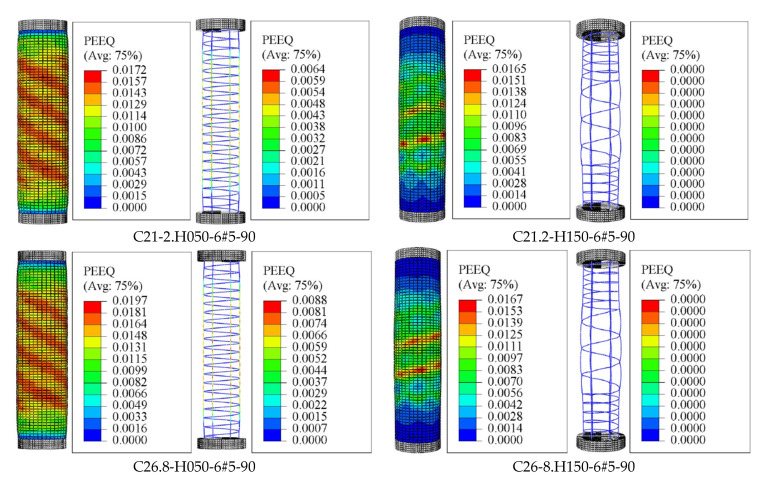
Finite element (FE) failure patterns: Considering the effect of varying amounts of GFRP confinement. Note: PEEQ = Equivalent plastic strain.

Figure 10 shows the results of selected simulated specimens with different concrete compressive strength and spiral spacing. These presented results were recorded when the damage in the concrete starts to occur (i.e., in this regard, the *d_c_* result is close to 0.7 for all test specimens). The results of Figure 10 declare that the effectiveness of confinement of FRP is usually limited by the buckling of longitudinal GFRP rebars. The results show that constructing columns with a larger spacing of the lateral reinforcing bars reduced the efficiency of concrete-core confinement and cause a global buckling for the longitudinal bars, i.e., C21-2-H150-6#5-90 and C26-8-H150-6#5-90, which exhibited no inelastic deformation in the reinforcing bars. The smaller spiral spacing reveals greater inelastic deformation which results in fracture of the longitudinal rebars, i.e., C21.2-H050-6#5-90 and C26.8-H050-6#5-90. Moreover, the GFRP spirals remained without any damage in all the experimental tested columns [7], and this can be seen from the results of Figure 9 and Figure 10.

**Figure 11:**
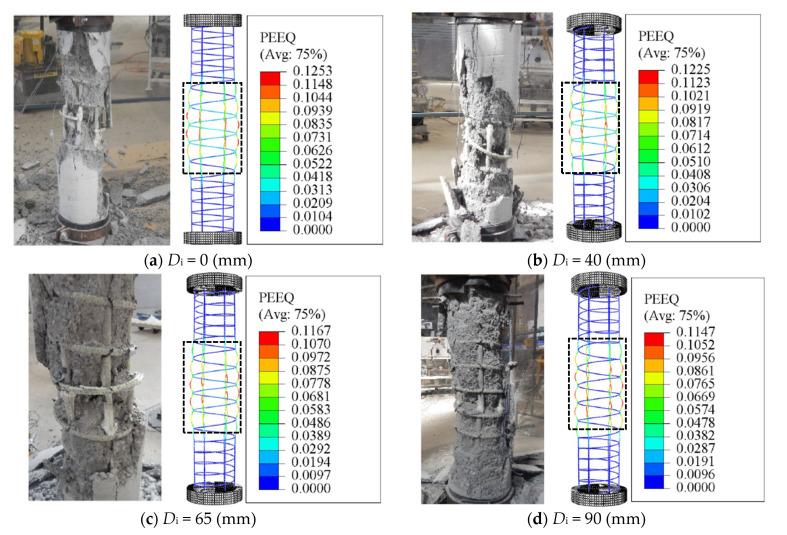
Experimental and finite element (FE) failure patterns of specimens: (**a**) C31.8-H100-6#5-00; (**b**) C31.8-H100-6#5-40; (**c**) C31.8-H100-6#5-65; (**d**) C31.8-H100-6#5-90: Considering the effect of different inner diameters. Note: PEEQ = Equivalent plastic strain.

**Figure 12:**
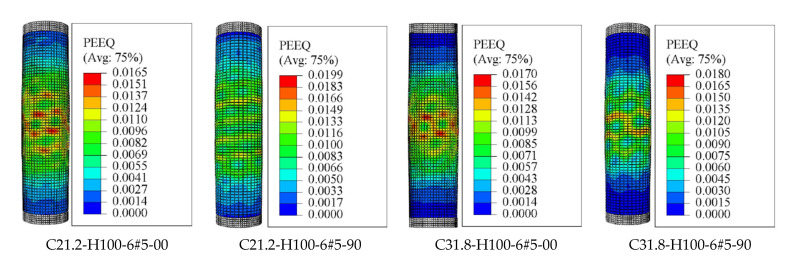
Finite element (FE) failure patterns: Considering the effect of different inner diameters.

However, the mechanism and extent of rupture of the longitudinal and transverse GFRP reinforcement varied among the columns constructed with different inner diameters [6]. It was concluded that increasing the inner-to-outer diameter ratio (*D_i_*/*D*) in the hollow columns changed the failure behavior from brittle to pseudo ductile. After spalling of concrete cover, the failure in the hollow columns with a *D_i_*/*D* ratio of 0.16 and 0.26 was initiated by the longitudinal and spiral GFRP reinforcement, while the failure of columns with a *D_i_*/*D* ratio of 0.36 was initiated by crushing of the hollow concrete-core. It is seen in Figure 11a,b that both the longitudinal and spiral GFRP reinforcement in C31.8-H100-6#5-00, C31.8-H100-6#5-40 are subjected to stress and damage. To explore this effects up to the failure of the specimens, the results of Figure 11 are corresponding to the final increment of the analysis (i.e., applied displacement = 50 mm). Figure 11c shows only the rupture of the longitudinal GFRP bars in C31.8-H100-6#5-90 with no rupture of GFRP spirals even after the bar rupture. Moreover, a clear comparison of the specimens’ results provided in Figure 11 with those of Figure 9b (spiral spacing = 50 mm) indicates that the damage in concrete along the entire column height is more obvious than the damage shown by the specimens in Figure 11 due to smaller amount of confinement (i.e., spirals with spacing equals to 100 mm). From Figure 11a–d, it can be also seen that the inelastic deformation (PEEQ) exhibited by the lateral and longitudinal GFRP reinforcement slightly decreases as the inner-hole diameter increases. This is because the rupture of the longitudinal GFRP rebars is dominant in the case of HCCS with a larger inner-hole diameter. However, Figure 12 shows a slight enhancement in the PEEQ observations for the tested HCCs, noting that these results were recorded when the damage in concrete was initiated (i.e., in this regard, the *d_c_* result is close to 0.7 for all test specimens). Generally, the HCCs with larger *D_i_*/*D* ratio values, i.e., C31.8-H100-6#5-90, showed better deformation capacity (represented by the PEEQ index) than those with a low *D_i_*/*D* ratio, i.e., C31.8-H100-6#5-00. This is due to the increased contribution of the GFRP bars to overall column stiffness and strength after spalling the concrete cover [6].

#### 4.3.2. Load-Strain Response

**Figure 13:**
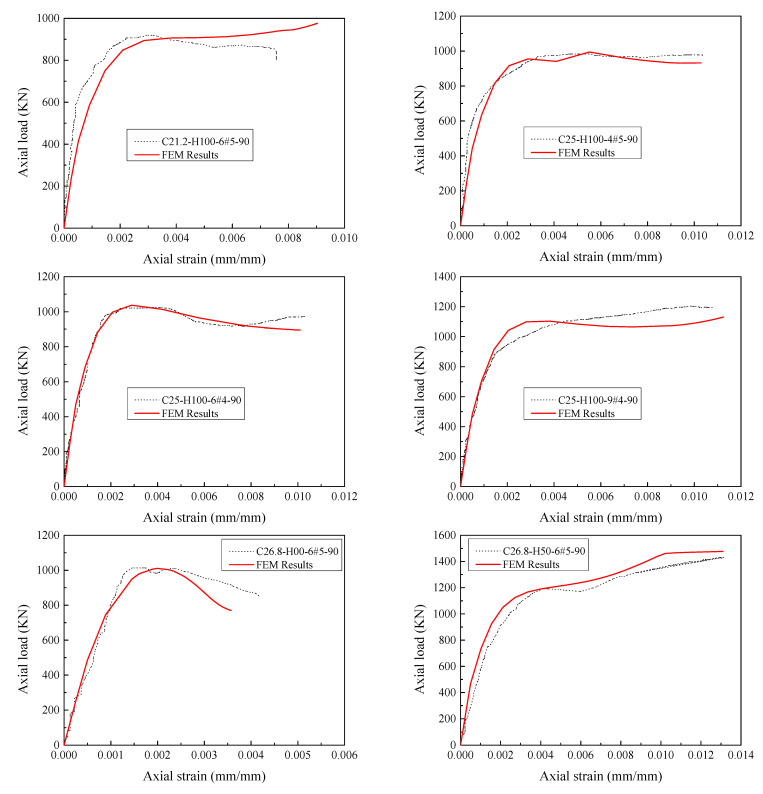

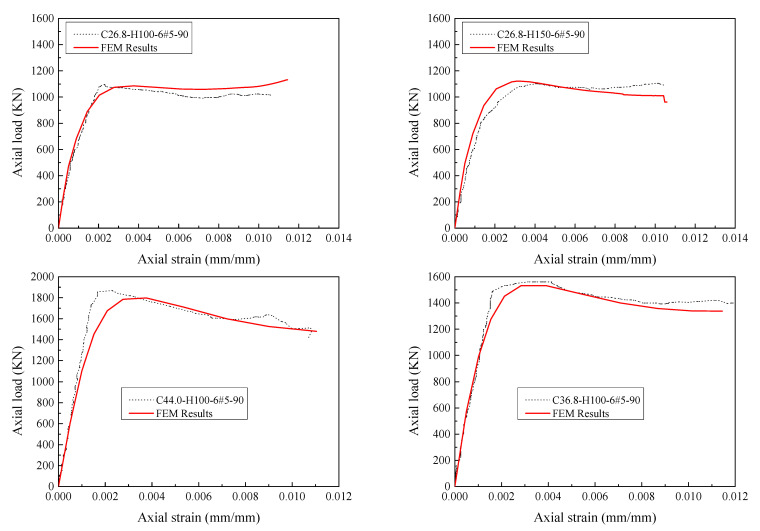
Comparison of tested and numerically obtained load-strain responses of HCCs.

**Figure 14:**
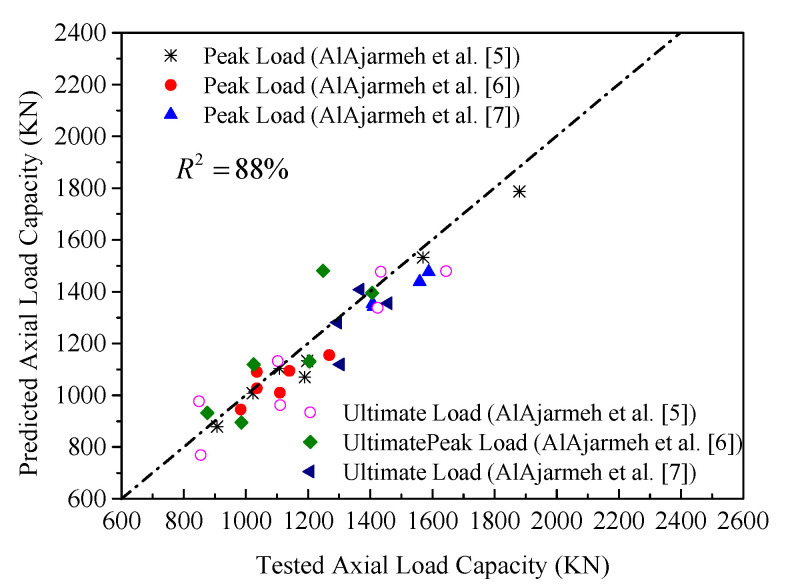
Comparison of tested and numerically obtained first and second axial peak capacities of HCCs.

Figure 13 represents the comparison of the complete load-strain responses of HCCs obtained from experimental and numerical results. The HCCs show different types of failure behavior: a softening behavior with one peak load (i.e., C26.8-H150-6#5-90) with a ductility factor of 1.5 and ductile behavior (i.e., C26.8-H50-6#5-90) with a two-peaks’ failure mode with a ductility factor of 2.1 comparing with the C26.8-H00-6#5-90 with no GFRP confinement. Overall, the axial load-strain responses of the finite element model agree well with the tested responses. Although there are some discrepancies between the experimental and numerical stress-strain responses (i.e., C25-H100-9#4-90), the comparison in Figure 14 showed a satisfactory correlation between the experimental and numerical axial capacities in pre-peak and post-peak zones. 

## 5. Artificial Neural Network (ANN) Technique

### 5.1. Model Development 

The ANN technique is used widely for many purposes such as classification, pattern recognition, and modeling. In particular, the use of ANN for predicting the compressive strength and strain of FRP-confined concrete has been studied (i.e., [52]). However, the use of ANN for GFRP HCCs has not yet been explored. In the aim of this, using the ANN toolbox provided in MATLAB R2020b [106], a model was developed to estimate the axial compressive load capacities for HCCs’ columns that exhibited softening and hardening behaviors. The number of experimental data used to construct and test the ANN model was 60 in total. In the development processes of the ANN model, an appropriate selection and reshaping of the input variables is a very important process. The axial load capacity of GFRP-reinforced HCCs should depend on the geometric dimensions and the properties of unconfined concrete and the confining material (i.e., GFRP spirals). As a result, the input variables, which appear to have significant effects on the axial load capacity [5,6,7], were taken into account with the following factors: (1) *λ_vb_* represented by Equation (21) is a non-dimensional factor to account for the effect of the longitudinal GFRP rebars, (2) *λ_lb_* represented by Equation (22) is a non-dimensional factor to account for the effect of the GFRP confinement by spirals, (3) *f_c_^’^* is the compressive strength of concrete, (4) *A_c_* is the effective concrete area without considering the longitudinal rebars, (5) *D_i_*/*D* is the inner-to-outer core diameter ratio.

(21) $$\lambda_{vb}=\frac{\rho_{e}f_{FRP}}{f_{c}^{’}}$$

(22) $$\lambda_{lb}=\frac{k_{e}\rho_{v}f_{FRP}}{f_{c}^{’}}$$

(23) $$k_{e}=\frac{A_{ce}}{A_{cc}}=\frac{\frac{\pi }{4}\left({\left(D_{s}-\frac{s^{’}}{4}\right)}^{2}-D_{i}^{2}\right)}{\frac{\pi }{4}\left(D_{s}^{2}-D_{i}^{2}\right)\left(1-\rho_{e}\right)}$$

The database was randomly divided into training (70%), validation (15%), and test (15%). Using one layer of hidden nodes based on previous suggestions [98], the optimum model parameters (i.e., the number of the hidden nodes, the rate of learning) were found by a proposed training approach. This can be performed by various available approaches, in which the network was trained with a set of random initial weights, hidden nodes’ numbers varying from 0 to 10, and the learning rate from 1 × 10^−2^ and 1 × 10^−1^. The Levenberg–Marquardt denoted by Trainlm [106,107] was selected as the training function. The performance function is *MSE*, and the transfer functions in both hidden and output layers are Pureline transfer functions. It is to be noted that the default transfer functions in the ANN toolbox are Tansig [106,107]. By transforming the data in the first and last ANN layers using the log function (Equations (24) and (25)) and choosing Pureline transfer functions, the ANN model reveals an acceptable performance:

(24) $$x={\left(\log_{10}[x_{1},x_{2}+1,x_{3},x_{4},x_{5}+1]\right)}^{T}$$

(25) $$y={\left(\log_{10}[y_{1},y_{2}]\right)}^{T}$$

**Figure 15:**
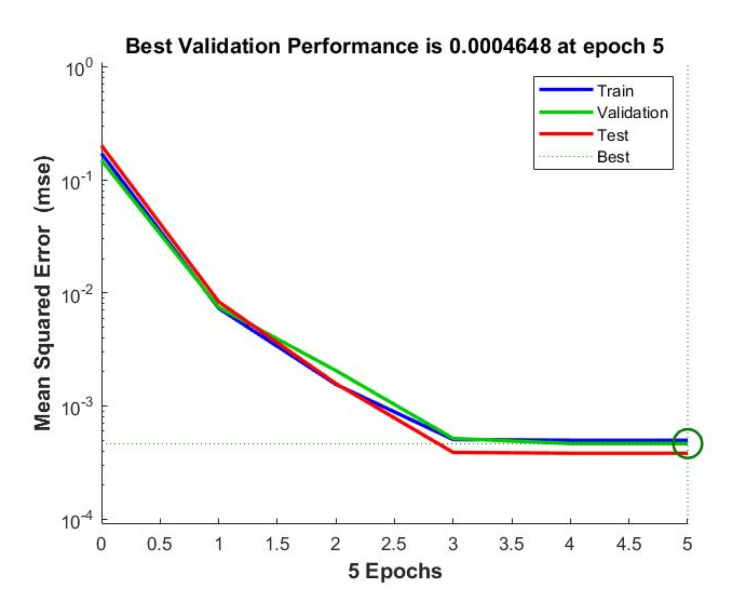
MSE errors in the train, validate, and test data.

Once the ANN model is built and the first and last data layers are chosen and normalized, the network can now be trained. Based on the overall model performance and the least mean square error achieved across a wide range of training parameters, the optimal number of hidden nodes was found to be 3 and the learning rate was close to the range of 0.005–0.01. Using these resulted parameters in a training approach, the most accurate results can then be obtained. Figure 15 clearly shows such a result for the train, validate, and test data, which have almost similar mean square errors. The predictions from the ANN model are practically generated following the Equations (26)–(32).

**Figure 16:**
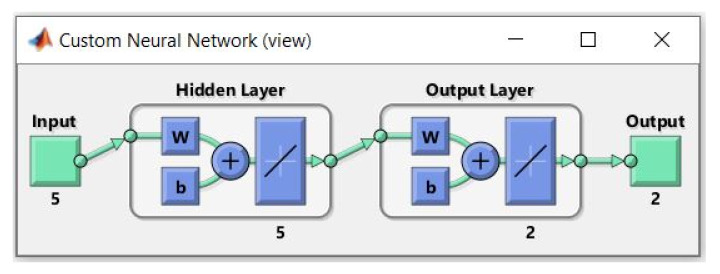
ANN architecture of the proposed first and second peak load capacities of HCCs.

(26) [(y−yminymax−ymin−0.5)×2]=10.^[w((x−xminxmax−xmin−0.5)×2)+a]

(27) $$w=w_{2}\times w_{1}$$

(28) $$a=w_{2}\times b_{1}+b_{2}$$

(29) $$w_{1}=\left[\begin{array}{ccccc} 0.0761 & -0.4257 & 0.7646\; & 1.0214 & 0.4346\\ -0.4925 & -1.0612 & 0.8116 & 0.0099 & -0.9388\\ -0.7003 & -1.1121 & -0.0234 & -0.8322 & 0.3971\\ -0.4369 & 0.4104 & 0.3133 & -0.4325 & 0.7582\\ 0.0827 & 0.0960 & -1.0293 & 0.6293 & -0.2225 \end{array}\right]$$

(30) $$w_{2}=\left[\begin{array}{ccccc} 0.3792 & 0.0417 & -0.4201 & -0.0688 & \;-0.6323\\ 0.4938 & -0.2168 & -0.8262 & -0.2278 & -0.9131 \end{array}\right]$$

(31) $$b_{1}=\left[\begin{array}{l} \begin{array}{c} -0.8411\\ -0.4814 \end{array}\\ -0.6591\\ 0.0193\\ -0.2438 \end{array}\right]$$

(32) $$b_{2}=\left[\begin{array}{c} -0.0381\\ -0.2204 \end{array}\right]$$

where *y* in Equation (26) represents the predictions; the inputs (*x*) and outputs (*y*) data of the ANN model are scaled using the minimum and maximum values provided in Table 5; *w*_1_ and *w*_2_ are essential findings of the ANN network. These contain the weights utilized between the *x* data and the hidden layer, and the hidden layer and the *y* data, respectively. The *b*_1_ and *b*_2_ are the matrices containing the bias of the hidden and output layers (see Figure 16). 

### 5.2. Performance of Proposed Axial Load Capacities 

**Figure 17:**
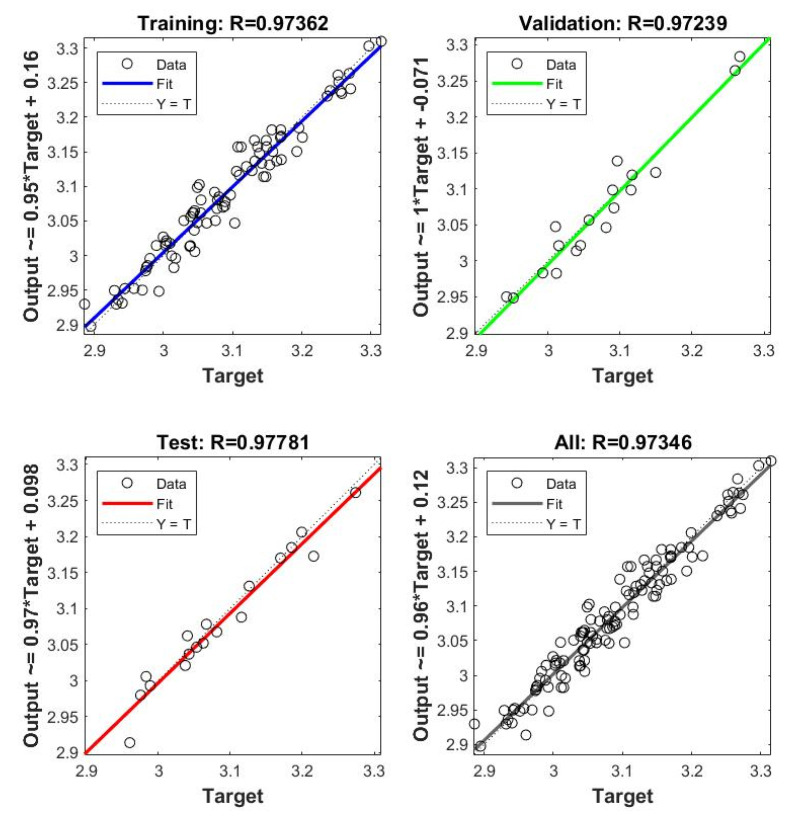
Overall performance of the proposed ANN model (N5-5-2).

**Figure 18:**
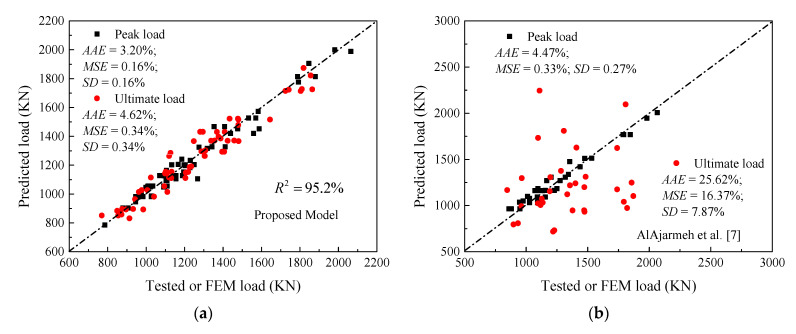
Performance of the proposed and existing models of HCCs: (**a**) Current ANN model; (**b**) AlAjarmeh et al.’s [7] analytical model.

The performance of the proposed ANN (N5-5-2) models of peak axial load capacities of GFRP-reinforced HCCs is verified by the database used to develop these models. The default generated figures from ANN toolbox [106,107] show excellent correlation. Figure 17 shows the overall accuracy of the proposed N5-5-2 model. Figure 18a,b provide comparisons of the ANN results with the test data. Due to the unavailability of models that consider GFRP-reinforced columns, only the AlAjarmeh et al.’s [7] model was, in this assessment, studied. Generally, these comparisons show that the estimated errors of the proposed ANN models were significantly lower than those of other methods. More specifically, the predictions of the first peak load resulted in less error by the proposed and existing models. However, the predicted peak loads of HCCs exhibiting a hardening failure model exhibited significant errors. This approves that the five variables of the ANN model (i.e., *λ_lb_*, *D_i_*/*D*) have a more significant effect on the second peak compared with their effects on the first peak load, which is well-taken into account by the ANN model with a high *R*^2^ of about 95.2%. 

### 5.3. Complete Axial Load-Strain Model 

**Figure 19:**
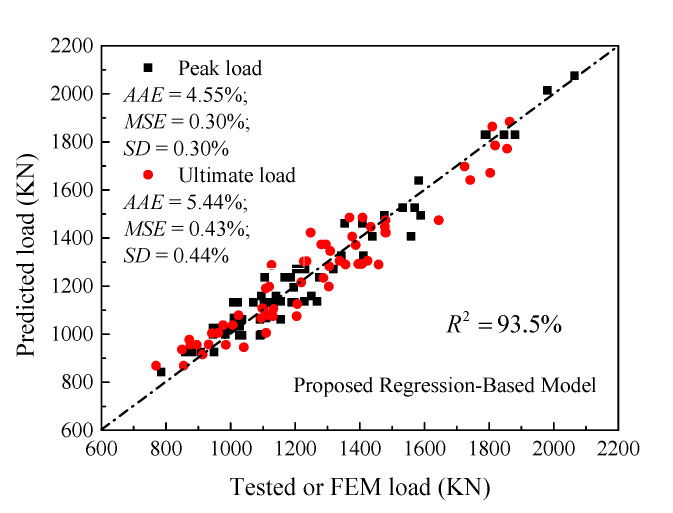
Performance of the proposed analytical model of HCCs.

First, the stress-strain monotonic curve drawn by Equation (33) is used as a control model for the tested GFRP-RC HCCs. The model details are shown in Equation (33). The first expression was firstly reported by Guo et al. [108] from results of tests on unreinforced rectangular concrete prisms, whereas the second one was used in many analytical studies:

(33) $$\left\{ \begin{array}{l} f_{c}=f_{cc1}[A\left(\frac{\varepsilon_{c}}{\varepsilon_{cc1}}\right)+\left(3-2A\right){\left(\frac{\varepsilon_{c}}{\varepsilon_{cc1}}\right)}^{2}+\left(A-2\right){\left(\frac{\varepsilon_{c}}{\varepsilon_{cc1}}\right)}^{3}]\;\;\;\;\;\;\;\;\;\;\varepsilon_{c}\leq \varepsilon_{cc1}\\ f_{c}=f_{cc1}+E_{2}(\varepsilon_{\mathrm{ccu}}-\varepsilon_{cc1})\;\;\;\;\;\;\;\;\;\;\;\;\;\;\;\;\;\;\;\;\;\;\;\;\;\;\;\;\;\;\;\;\;\;\;\;\;\;\;\;\;\;\;\;\;\;\;\;\;\;\;\;\;\;\;\;\;\;\;\;\;\varepsilon_{cc1}\leq \varepsilon_{c}\leq \varepsilon_{ccu} \end{array}\right.$$

where *E*_2_ (MPa) is the slope of the post-peak linear portion of the stress-strain response and determined by Equation (34); the strain at the first peak *ε_cc_*_1_ and second peak *ε_ccu_* can be obtained from Equations (4) and (19), respectively. The shape parameter A, which controls the polynomial portion, is derived from the boundary condition of *d_σc_*/*d_εc_* = *E_c_* at *ε_c_* = 0. The parameter *A* can be obtained as *A = E_c_*/*E_p_* by substituting the boundary value in Equation (33):

(34) $$E_{2}=\frac{f_{\mathrm{ccu}}-f_{cc1}}{\varepsilon_{\mathrm{ccu}}-\varepsilon_{cc1}}$$

where *E_c_* (MPa) is predicted using Equation (1); *E_p_ = f_cc_*_1_/*ε_cc_*_1_ (MPa) is the second modulus at the peak point; the terms *f_cc_*_1_ and *f_ccu_* are calculated by dividing the first and second peak loads obtained using the ANN model over the effective concrete area without considering the longitudinal rebars. The *f_cc_*_1_ and *f_ccu_* can be also predicted using Equations (35) and (36), additionally proposed in the present work with a correlation factor *R*^2^ of about 95.1 and 91.4% (see Figure 19). Overall, the comparison between the predictions made by this model and the ANN model as well as the test results shows that the accuracy is almost typical (ANN model’s *R*^2^ = 95.2% and *RA* model’s *R*^2^ = 93.5% (on average)).

(35) $$\begin{array}{l} p_{n1}=\alpha_{1}f_{c}^{’}\left(A_{g}-A_{FRP}\right)+0.0032E_{FRP}A_{FRP}\\ \left(\alpha_{1}=0.713+37\times 10^{-4}f_{c}^{’}\geq 0.798\right) \end{array}$$

(36) $$p_{n2}=A_{c}f_{c}^{’}\left(0.41+0.07{\left(\lambda_{vb}\right)}^{2.65}+0.91\exp(\lambda_{lb}{}^{0.61})\exp{\left(1+\frac{D_{i}}{D}\right)}^{-1.24}\right){\left(1+\frac{D_{i}}{D}\right)}^{0.23}$$

**Figure 20:**
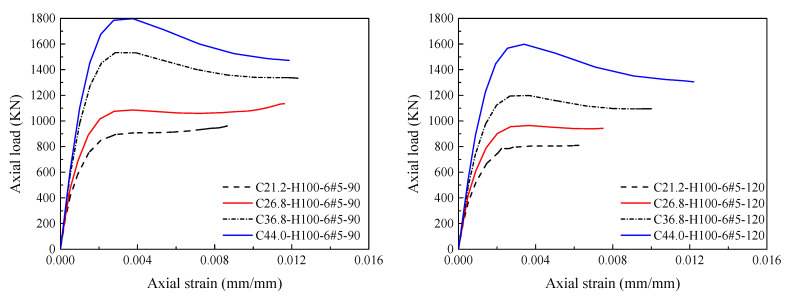
Effect of varying compressive strengths of concrete on the load-strain response of HCCs.

**Figure 21:**
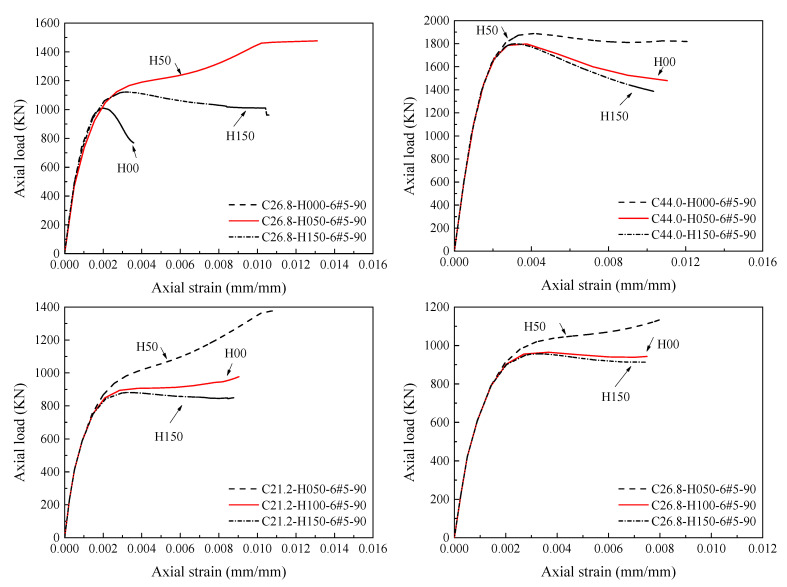
Effect of varying confinement ratios of GFRP spirals on the load-strain response of HCCs.

**Figure 22:**
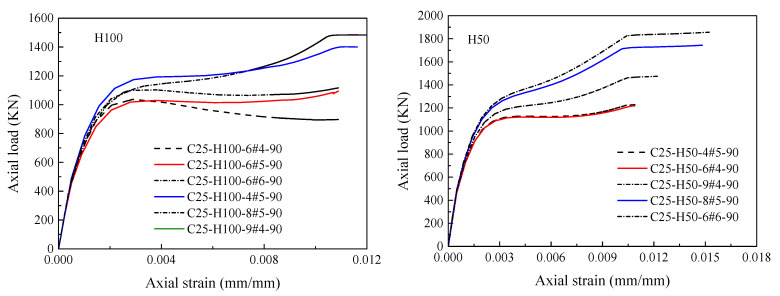
Effect of different amounts of longitudinal reinforcement on the load-strain response of HCCs.

**Figure 23:**
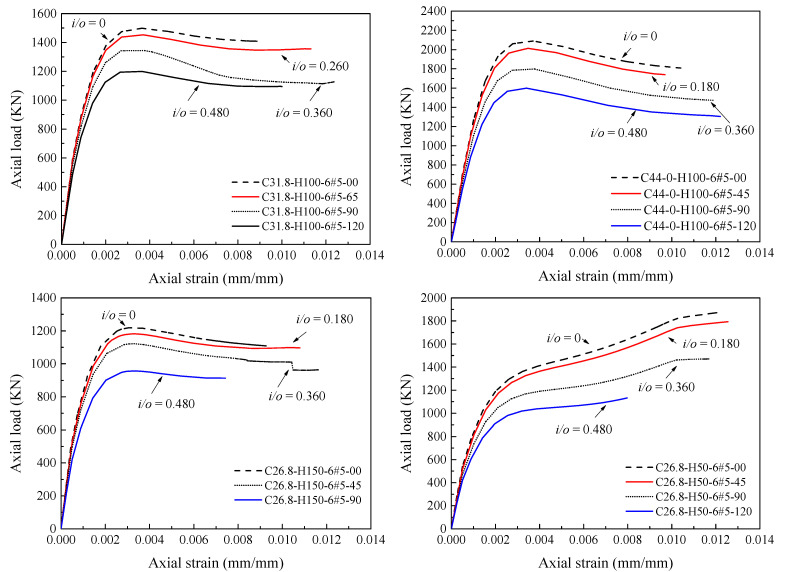
Effect of different inner-to-outer (*D_i_*/*D*) diameter ratios on the load-strain response of HCCs.

**Figure 24:**
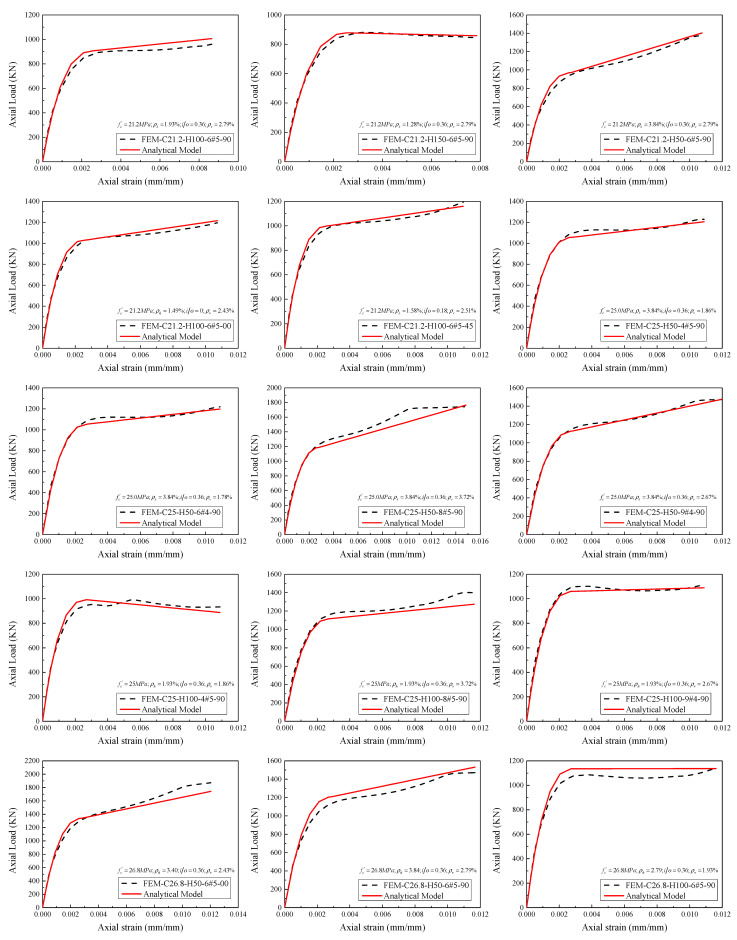

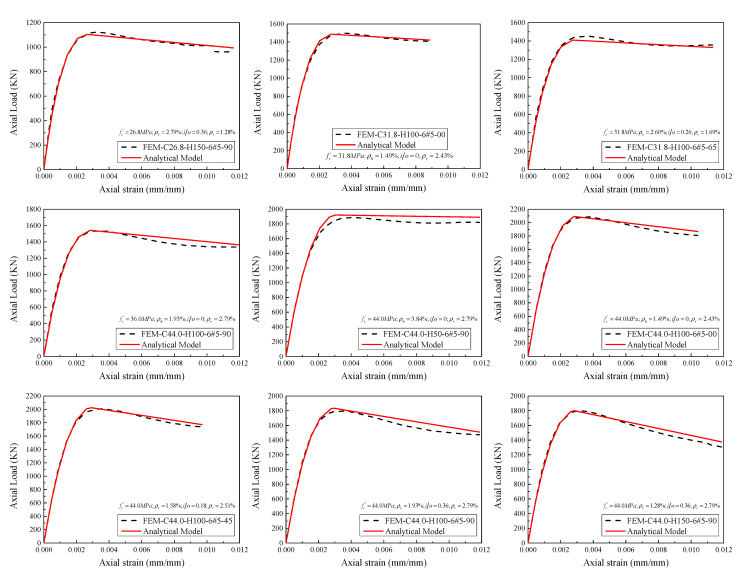
Comparison between predictions of proposed concrete axial load-axial strain curves, and experimental and numerical results.

After predicting the stress-strain relationship of HCCs by Equations (33) and (34), the load-strain curve can be generated. To evaluate the proposed model in terms of significant effects of the key parameters considered in the present paper as respectively demonstrated in Figure 20, Figure 21, Figure 22 and Figure 23, comparisons between load-strain responses of the present model with selected experimental results of specimens with various test parameters from both the experiments provided in [5,6,7] and the FE simulations are provided in Figure 24. Inspection of the comparisons with the results demonstrates that the model can capture well the major features of the response such as the axial load capacities *p_n_*_1_ and *p_n_*_2_. Generally, the pre-peak and post-peak regions of the simulated response are also described in a good way. 

## 6. Proposed Minimum Thresholds for Acceptable Performance 

### 6.1. Thresholds of Inner-to-Outer Core Diameter (D_i_/D) Ratios 

**Figure 25:**
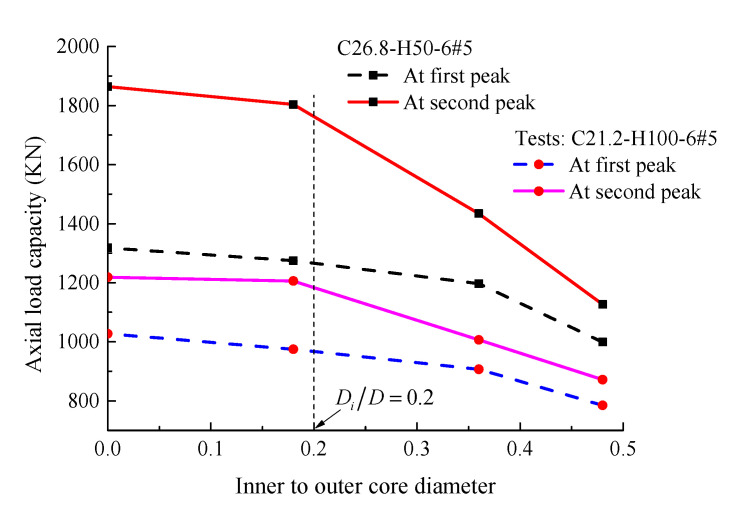
Effect of increasing the *D_i_*/*D* ratio on the axial load capacity.

**Figure 26:**
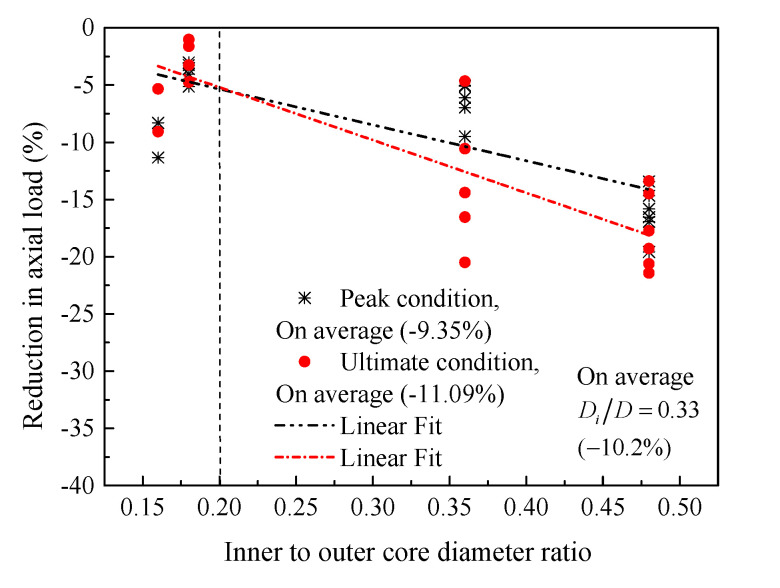
Relationship between varying *D_i_*/*D* ratios and reduction in peak loads.

Tests [6] have confirmed that the hollow columns (*D_i_*/*D* > 0) failed at a lower load than the solid column (*D_i_*/*D* = 0) due to the reduced effective area. To gain sufficient understanding about this issue, Figure 25 presents the results of specimens C26.8-H50-6#5 and C21.2-H100-6#5 selected from Table 2. It is seen that when the *D_i_*/*D* ratio is being less than 0.2, the difference in concrete compressive loads of the hollow and solid columns is insignificant. As stated in previous discussions, the failure of the HCCs having an inner- to-outer concrete-core diameter ratio of 0.16 and 0.26 was initiated by the longitudinal reinforcement and spiral, while the failure of the HCCs having an inner-to-outer concrete-core diameter ratio of 0.36 was initiated by crushing of the inner concrete core. This also demonstrated that both the longitudinal and lateral GFRP reinforcement contributed to the load enhancement when the *D_i_*/*D* ratio is less than 0.26 since they appear to be subjected to stress up to column failure, while only the longitudinal reinforcement contributed to the improvement of the load capacity for columns with larger *D_i_*/*D* ratios (i.e., *D_i_*/*D* = 0.36), which ultimately resulted in lower loads than the solid sections. The hollow concrete-core C26.8-H50-6#5-120 with a *D_i_*/*D* ratio of 0.48 exhibited 24.2% and 39.6% lower axial capacities at the first and second peak loading conditions than the solid specimen C26.8-H50-6#5-00, respectively. The effect of increasing the *D_i_*/*D* ratio is more pronounced on the second peak load at a higher *D_i_*/*D* ratio. It is suggested in Figure 26 that the averaged *D_i_*/*D* ratio for all tests of the present paper that causes an averaged reduction of almost 10% in the load-carrying capacity is equal to 0.33. The reduced load can be compensated by sufficient confinement of GFRP as discussed next.

### 6.2. Minimum Amount of FRP for Adequate Confinement

A designed column needs a minimum amount of reinforcement materials for sufficient confinement [86,87,88,89,90,91,92,93,94,95,96,97,98,99,100,101,109]. In these cases, if the compression load ratio (i.e., *p_n_*_2_/*p_n_*_1_) is greater than one, the resulting threshold represents the sufficiently confined concrete [86,87,88,89,90,91,92,93,94,95,96,97,98,99,100,101,109]. Based on an analytical investigation by Pham and Hadi [109] on FRP-confined circular and non-circular columns under concentric compression, the minimum threshold value of effective confinement stress ratio was suggested to be 0.15. For GFRP-reinforced hollow concrete-core columns, the test results with different geometric and material characteristics were studied. 

**Figure 27:**
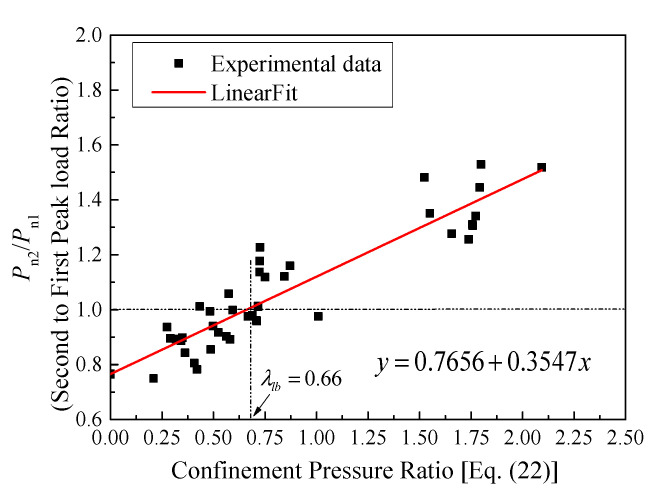
Relationship between confinement pressure ratio and confined load ratio.

The response between the effective confining pressure ratio and the confined axial load ratio is given in Figure 27. Based on an averaged trend-line made between the experimental, FEM, and parametric data, when the load ratio is equal to 1.0, then the confinement pressure ratio (Equation (22)) should be over 0.66 for the present tests, and such a threshold is larger than that of FRP-confined circular columns due to the reduced effects caused by different material properties and that of the tensile strength of the bent GFRP rebars. 

## 7. Conclusions 

The present work aims to investigate the structural behavior of hollow concrete-core columns reinforced with GFRP bars by performing detailed numerical simulation, analytically provided in this work, and the key conclusions are summarized below. 

During the calibration of the FEM model, it was found that the dilation angle of 30° and the concrete’s viscosity parameter of 5 × 10^−5^ gave a good performance while using the concrete damage plasticity model (CDPM). Moreover, the mesh size of 15 mm for C3D8R elements of concrete and 10 mm for both C3D8R and T3D2R elements of end plates and GFRP bars presented good performance for predicting load-strain behavior and failure patterns of HCCs.

- The proposed FEM model captured the structural response of GFRP-reinforced HCCs with high accuracy. The average correlated *R*^2^ value between the available testing results and numerical data was 88% for both the peak and ultimate loads.
- The failure modes of the HCCs were numerically obtained and compared with the tested ones. Both the experimentally tested and numerically obtained patterns were in a good match. The influence of varying test parameters (i.e., amount of longitudinal and lateral reinforcement) on these patterns can be seen.
- To estimate the maximum axial load capacity of GFRP-RC HCCs, the contribution of the reinforcement is necessary. The authors proposed a new model for predicting the peak axial load capacity of GFRP-RC HCCs based on a database of 60 FRP-reinforced columns with *R*^2^ = 0.95.
- To capture the overall stress-strain response of FRP-reinforced columns, artificial neural network (ANN) models are proposed to estimate both the axial compressive loads at the first and second peak conditions. The effects of different amounts of longitudinal reinforcement, volumetric radios of GFRP confinement, inner-to-outer diameter ratios, and compressive strengths of the standard cylinder were all considered in the model. Besides, several RA (Regression Analytical) models to predict the different components of the axial strength and strains have been introduced and some of them are compared with the ANN models. For this purpose, the proposed ANN models have been successfully applied using transformed easy-to-use equations rather than using their complex computational models. The predicted results of the proposed analytical model agree well with the tested and numerically obtained results. They yield better results with marginal errors as compared with the existing analytical models.
- A design-oriented stress-strain model that can capture the softening and hardening behaviors of GFRP-reinforced HCCs was suggested. The model features and the test parameters appeared to be accurately considered when the experimental and analytical load-strain responses matched closely.
- Based on parametric, experimental, and numerical data, the sufficiently confined concrete threshold of FRP-reinforced columns was proposed to be at least 0.66. This guarantees a safe and good design for HCCs resulting in a hardening behavior.

## Figures and Tables

**Table 1:** Summary of the design equations available in the literature for FRP-RC columns.

Reference	Proposed Equation	FRP	Predicted Condition	Estimated Errors		
				AAE (%)	MSE (%)	SD (%)
CSA-S806-12 [60]	$p_{n1}=0.85f_{c}^{'}\left(A_{g}-A_{FRP}\right)$	–	Peak Ultimate	15.74 18.01	2.78 5.27	0.31 2.78
ACI318-14 [62]	$p_{n1}=0.85f_{c}^{'}\left(A_{g}-A_{FRP}\right)$	–	Peak Ultimate	15.74 18.01	2.78 5.27	0.31 2.78
Tobbi et al. [3]	$p_{n1}=0.85f_{c}^{'}\left(A_{g}-A_{FRP}\right)+0.35f_{FRP}A_{FRP}$	G	Peak Ultimate	28.10 28.65	9.06 11.07	1.22 4.19
Afifi et al. [4]	$p_{n1}=0.85f_{c}^{'}\left(A_{g}-A_{FRP}\right)+0.35f_{FRP}A_{FRP}$	G	Peak Ultimate	28.10 28.65	9.06 11.07	1.22 4.19
Afifi et al. [65]	$p_{n1}=0.85f_{c}^{'}\left(A_{g}-A_{FRP}\right)+0.25f_{FRP}A_{FRP}$	C	Peak Ultimate	15.57 19.92	3.10 5.55	0.70 3.61
Mohamed et al. [66]	$p_{n1}=0.85f_{c}^{'}\left(A_{g}-A_{FRP}\right)+0.002E_{FRP}A_{FRP}$	G	Peak Ultimate	5.44 13.75	0.43 3.09	0.31 3.00
Maranan et al. [67]	$p_{n1}=0.9f_{c}^{'}\left(A_{g}-A_{FRP}\right)+0.002E_{FRP}A_{FRP}$	G	Peak Ultimate	4.77 14.70	0.35 3.25	0.34 3.34
Hadhood et al. [63]	$p_{n1}=\alpha_{1}f_{c}^{'}\left(A_{g}-A_{FRP}\right)+0.0035E_{FRP}A_{FRP}\left(\alpha_{1}=0.85-0.0015f_{c}^{'}\geq 0.67\right)$	C	Peak Ultimate	6.02 13.04	0.48 2.77	0.49 2.87
Hadhood et al. [63]	$p_{n1}=0.85f_{c}^{'}\left(A_{g}-A_{FRP}\right)+0.003E_{FRP}A_{FRP}$	C	Peak Ultimate	5.31 14.16	0.43 3.09	0.38 3.16
Hadhood et al. [64]	$p_{n1}=0.85f_{c}^{'}\left(A_{g}-A_{FRP}\right)+0.0024E_{FRP}A_{FRP}$	C	Peak Ultimate	4.99 13.77	0.34 3.00	0.34 3.06
Xue et al. [68]	$p_{n1}=0.85f_{c}^{'}\left(A_{g}-A_{FRP}\right)+0.002E_{FRP}A_{FRP}$	G	Peak Ultimate	5.44 13.75	0.43 3.09	0.31 3.00

Note: *A_FRP_* = area of FRP longitudinal reinforcement; *A_g_* = gross area of column section; *E_FRP_* = tensile Young’s modulus of the FRP bars; *f_c_^’^* = compressive strength of unconfined concrete; *f_FRP_* = FRP tensile strength; *P_n_*_1_ = nominal capacity corresponding to first peak load.

**Table 2:** Test database of GFRP-RC columns.

Coded Columns with Geometry and Concrete Type				Reinforcement Details			Longitudinal Reinforcement			Hoop Reinforcement				Key Experimental Results	
*Code*	*D* (mm)	*D_i_* (mm)	*f_c_^’^* (MPa)	*ρ_FRP_* (%)	*Reinforcement* *Hoop*/*Longitudinal*		*f_FRP_* (MPa)	*E_FRP_* (GPa)	*ε_FRP_* (%)	*f_FRP_* (MPa)	*E_FRP_* (MPa)	*ε_FRP_* (%)	*P_n_*_1_ (KN)	*P_n_*_2_ (KN)	*ε_cu_* (%)
AlAjarmeh et al. [7]															
C26.8-H00-6#5-90	250	90	26.8	0.000	-	6ϕ15.9	1237	60,000	2.1	1315	62,500	2.3	1022.0	854.6	0.387
C26.8-H50-6#5-90	250	90	26.8	3.840	9.5ϕ50	6ϕ15.9	1237	60,000	2.1	1315	62,500	2.3	1197.0	1434.0	1.233
C26.8-H100-6#5-90	250	90	26.8	1.930	9.5ϕ100	6ϕ15.9	1237	60,000	2.1	1315	62,500	2.3	1189.0	1102.0	1.110
C26.8-H150-6#5-90	250	90	26.8	1.280	9.5ϕ150	6ϕ15.9	1237	60,000	2.1	1315	62,500	2.3	1108.0	1110.0	1.102
C21.2-H100-6#5-90	250	90	21.2	1.930	9.5ϕ100	6ϕ15.9	1237	60,000	2.1	1315	62,500	2.3	907.0	1006.7	0.743
C36.8-H100-6#5-90	250	90	36.8	1.930	9.5ϕ100	6ϕ15.9	1237	60,000	2.1	1315	62,500	2.3	1570.0	1424.0	1.347
C44.0-H100-6#5-90	250	90	44.0	1.930	9.5ϕ100	6ϕ15.9	1237	60,000	2.1	1315	62,500	2.3	1880.0	1644.0	1.128
AlAjarmeh et al. [5]															
C25-H100-6#4-90	250	90	25.0	1.930	9.5ϕ100	6ϕ12.7	1282	61,300	2.1	1315	62,500	2.3	1035.3	985.1	1.058
C25-H100-6#5-90	250	90	25.0	1.930	9.5ϕ100	6ϕ15.9	1237	60,500	2.1	1315	62,500	2.3	1109.2	1024.4	1.069
C25-H100-6#6-90	250	90	25.0	1.930	9.5ϕ100	6ϕ19.1	1270	60,500	2.1	1315	62,500	2.3	1140.0	1247.9	1.148
C25-H100-4#5-90	250	90	25.0	1.930	9.5ϕ100	4ϕ15.9	1237	60,500	2.1	1315	62,500	2.3	983.3	875.5	1.062
C25-H100-8#5-90	250	90	25.0	1.930	9.5ϕ100	8ϕ15.9	1237	60,500	2.1	1315	62,500	2.3	1267.9	1406.1	1.106
C25-H100-9#4-90	250	90	25.0	1.930	9.5ϕ100	9ϕ12.7	1282	61,300	2.1	1315	62,500	2.3	1035.0	1204.2	1.077
AlAjarmeh et al. [6]															
C31.8-H100-6#5-00	250	0	31.8	1.490	9.5ϕ100	6ϕ15.9	1237	60,000	2.1	1315	62,500	2.3	1588.0	1368.0	-
C31.8-H100-6#5-40	250	40	31.8	1.560	9.5ϕ100	6ϕ15.9	1237	60,000	2.1	1315	62,500	2.3	1408.0	1295.0	-
C31.8-H100-6#5-65	250	65	31.8	1.690	9.5ϕ100	6ϕ15.9	1237	60,000	2.1	1315	62,500	2.3	1559.0	1458.0	-
C31.8-H100-6#5-90	250	90	31.8	1.930	9.5ϕ100	6ϕ15.9	1237	60,000	2.1	1315	62,500	2.3	1411.0	1304.0	-
Finite Element (FE) experiments [49]															
C26.8-H00-6#5-90	250	90	26.8	0.000	-	6ϕ15.9	1237	60,000	2.1	1315	62,500	2.3	1007.8	769.2	0.357
C26.8-H50-6#5-90	250	90	26.8	3.840	9.5ϕ50	6ϕ15.9	1237	60,000	2.1	1315	62,500	2.3	1133.2	1477.5	1.310
C26.8-H100-6#5-90	250	90	26.8	1.930	9.5ϕ100	6ϕ15.9	1237	60,000	2.1	1315	62,500	2.3	1070.2	1132.3	1.143
C26.8-H150-6#5-90	250	90	26.8	1.280	9.5ϕ150	6ϕ15.9	1237	60,000	2.1	1315	62,500	2.3	1103.6	962.0	1.059
C21.2-H100-6#5-90	250	90	21.2	1.930	9.5ϕ100	6ϕ15.9	1237	60,000	2.1	1315	62,500	2.3	879.5	976.7	0.903
C36.8-H100-6#5-90	250	90	36.8	1.930	9.5ϕ100	6ϕ15.9	1237	60,000	2.1	1315	62,500	2.3	1532.4	1337.6	1.144
C44.0-H100-6#5-90	250	90	44.0	1.930	9.5ϕ100	6ϕ15.9	1237	60,000	2.1	1315	62,500	2.3	1787.4	1479.8	1.104
C25-H100-6#4-90	250	90	25.0	1.930	9.5ϕ100	6ϕ12.7	1282	61,300	2.1	1315	62,500	2.3	1027.3	895.2	1.010
C25-H100-6#5-90	250	90	25.0	1.930	9.5ϕ100	6ϕ15.9	1237	60,500	2.1	1315	62,500	2.3	1010.0	1119.2	1.113
C25-H100-6#6-90	250	90	25.0	1.930	9.5ϕ100	6ϕ19.1	1270	60,500	2.1	1315	62,500	2.3	1094.6	1480.6	1.315
C25-H100-4#5-90	250	90	25.0	1.930	9.5ϕ100	4ϕ15.9	1237	60,500	2.1	1315	62,500	2.3	945.2	932.2	1.029
C25-H100-8#5-90	250	90	25.0	1.930	9.5ϕ100	8ϕ15.9	1237	60,500	2.1	1315	62,500	2.3	1155.0	1395.1	1.270
C25-H100-9#4-90	250	90	25.0	1.930	9.5ϕ100	9ϕ12.7	1282	61,300	2.1	1315	62,500	2.3	1090.6	1130.7	1.124
C25-H50-6#4-90	250	90	25.0	3.840	9.5ϕ50	6ϕ12.7	1282	61,300	2.1	1315	62,500	2.3	1091.4	1226.5	1.194
C25-H50-6#6-90	250	90	25.0	3.840	9.5ϕ50	6ϕ19.1	1270	60,500	2.1	1315	62,500	2.3	1249.7	1855.8	1.512
C25-H50-4#5-90	250	90	25.0	3.840	9.5ϕ50	4ϕ15.9	1237	60,500	2.1	1315	62,500	2.3	1094.7	1234.9	1.199
C25-H50-8#5-90	250	90	25.0	3.840	9.5ϕ50	8ϕ15.9	1237	60,500	2.1	1315	62,500	2.3	1228.0	1741.8	1.456
C25-H50-9#4-90	250	90	25.0	3.840	9.5ϕ50	9ϕ12.7	1282	61,300	2.1	1315	62,500	2.3	1155.2	1478.9	1.323
C31.8-H100-6#5-00	250	0	31.8	1.490	9.5ϕ100	6ϕ15.9	1237	60,000	2.1	1315	62,500	2.3	1476.0	1408.6	0.940
C31.8-H100-6#5-40	250	40	31.8	1.560	9.5ϕ100	6ϕ15.9	1237	60,000	2.1	1315	62,500	2.3	1353.5	1280.8	0.977
C31.8-H100-6#5-65	250	65	31.8	1.690	9.5ϕ100	6ϕ15.9	1237	60,000	2.1	1315	62,500	2.3	1438.6	1355.9	1.097
C31.8-H100-6#5-90	250	90	31.8	1.930	9.5ϕ100	6ϕ15.9	1237	60,000	2.1	1315	62,500	2.3	1342.8	1119.4	1.116
C26.8-H50-6#5-00	250	0	26.8	3.402	9.5ϕ50	6ϕ15.9	1237	60,000	2.1	1315	62,500	2.3	1317.9	1864.2	1.175
C26.8-H50-6#5-45	250	45	26.8	3.486	9.5ϕ50	6ϕ15.9	1237	60,000	2.1	1315	62,500	2.3	1275.0	1803.6	1.305
C26.8-H150-6#5-00	250	0	26.8	0.842	9.5ϕ150	6ϕ15.9	1237	60,000	2.1	1315	62,500	2.3	1203.1	1109.2	0.921
C26.8-H150-6#5-45	250	45	26.8	0.926	9.5ϕ150	6ϕ15.9	1237	60,000	2.1	1315	62,500	2.3	1166.7	1097.9	1.015
C21.2-H100-6#5-00	250	0	21.2	1.492	9.5ϕ100	6ϕ15.9	1237	60,000	2.1	1315	62,500	2.3	1027.4	1218.7	1.134
C21.2-H100-6#5-45	250	45	21.2	1.576	9.5ϕ100	6ϕ15.9	1237	60,000	2.1	1315	62,500	2.3	974.9	1206.2	1.224
C21.2-H50-6#5-90	250	90	21.2	3.840	9.5ϕ50	6ϕ15.9	1237	60,000	2.1	1315	62,500	2.3	948.5	1376.3	1.077
C21.2-H150-6#5-90	250	90	21.2	1.280	9.5ϕ150	6ϕ15.9	1237	60,000	2.1	1315	62,500	2.3	860.1	849.2	0.876
C44.0-H100-6#5-00	250	0	44.0	1.492	9.5ϕ100	6ϕ15.9	1237	60,000	2.1	1315	62,500	2.3	2064.5	1810.1	1.031
C44.0-H100-6#5-45	250	45	44.0	1.576	9.5ϕ100	6ϕ15.9	1237	60,000	2.1	1315	62,500	2.3	1981.1	1724.2	1.055
C44.0-H50-6#5-90	250	90	44.0	3.840	9.5ϕ50	6ϕ15.9	1237	60,000	2.1	1315	62,500	2.3	1846.5	1818.8	1.206
C44.0-H150-6#5-90	250	90	44.0	1.280	9.5ϕ150	6ϕ15.9	1237	60,000	2.1	1315	62,500	2.3	1792.3	1387.1	1.033
C26.8-H100-6#5-00	250	0	26.8	1.492	9.5ϕ100	6ϕ15.9	1237	60,000	2.1	1315	62,500	2.3	1229.7	1308.4	1.036
C26.8-H100-6#5-45	250	45	26.8	1.576	9.5ϕ100	6ϕ15.9	1237	60,000	2.1	1315	62,500	2.3	1185.6	1287.4	1.137
C44.0-H100-6#5-120	250	120	44.0	2.361	9.5ϕ100	6ϕ15.9	1237	60,000	2.1	1315	62,500	2.3	1582.5	1305.1	1.220
C31.8-H100-6#5-120	250	120	31.8	2.361	9.5ϕ100	6ϕ15.9	1237	60,000	2.1	1315	62,500	2.3	1194.9	1094.3	1.000
C26.8-H00-6#5-45	250	45	26.8	0.000	-	6ϕ15.9	1237	60,000	2.1	1315	62,500	2.3	1105.8	1041.0	0.357
C26.8-H50-6#5-120	250	120	26.8	4.271	9.5ϕ50	6ϕ15.9	1237	60,000	2.1	1315	62,500	2.3	999.0	1126.8	0.783
C26.8-H100-6#5-120	250	120	26.8	2.361	9.5ϕ100	6ϕ15.9	1237	60,000	2.1	1315	62,500	2.3	956.1	942.4	0.744
C26.8-H150-6#5-120	250	120	26.8	1.711	9.5ϕ150	6ϕ15.9	1237	60,000	2.1	1315	62,500	2.3	945.4	913.2	0.737
C21.2-H100-6#5-120	250	120	21.2	2.361	9.5ϕ100	6ϕ15.9	1237	60,000	2.1	1315	62,500	2.3	785.2	872.0	0.929

**Table 3:** Properties of finite element (FE) simulations.

Property	Value
Mesh size of concrete material (mm)	15/Element Shape: Hex-Structured
Mesh size of steel end plates (mm)	10/Element Shape: Hex-Sweep
Mesh size of GFRP rebars and spirals (mm)	10
Minimum step size	1 × 10^−8^
Initial step size	0.005
Maximum step size	0.01
Concrete and steel end plate element type	Standard C3D8R/Geometric Order: Linear/Family: 3D Stress
GFRP rebars and spiral element type	Standard T3D2/Geometric Order: Linear/Family: Truss
Interaction constraint of FRP reinforcement to concrete	Embedded Region
Applied displacement (mm)	50
Step-1 type	Static, General
Equation solver	Direct
Automatic stabilization	Use damping factors from previous general steps

**Table 4:** Plasticity parameters of concrete for CDP.

Parameter	Value
Dilatation angle (*ψ*), Degrees	30
Plastic potential eccentricity (*ε*)	0.1
*σ_bo_*/*σ_co_* [77]	1.5(*f_c_^’^*^’^)^−0.075^
The shape factor of yielding surface (*K*_c_)	0.6667
Viscosity parameter (𝜇)	5 × 10^5^

**Table 5:** Data to scale the inputs’ data.

Input/Output		Maximum	Minimum	Mean	Standard Deviation
*λ* *_vb_*	–	2.04	0.68	1.19	0.35
*λ* *_lb_*	–	2.09	0.00	0.68	0.51
*f_c_^’^*	MPa	44.00	21.20	26.80	6.57
*A_c_*	mm^2^	47,916.14	36,601.86	41,551.86	3138.02
*D_i_*/*D*	(mm/mm)	0.48	0.00	0.36	0.14
*p_n_*_1_	Kn	2064.49	785.21	1155.13	291.75
*p_n_*_2_	Kn	1864.16	769.17	1241.42	279.56

## Data Availability

The data presented in this study are available on request from the corresponding author.

## Abbreviations

*A_c_*	concrete section area (without taking into account the longitudinal GFRP bars) (*A_g_-A_FRP_*) (mm^2^):
*A_cc_*	concrete-core area (without taking into account the longitudinal GFRP bars) (*A_core_-A_FRP_*) (mm^2^);
*A_ce_*	area of the concrete core (mm^2^);
*A_g_*	gross cross-sectional area of concrete column (mm^2^);
*A_core_*	effective core area denoted by the center-to-center spacing of the GFRP spirals (mm^2^);
*A_FRP_*	the total cross-sectional area of the vertical GFRP bars (mm^2^);
*A_h_*	the cross-sectional area of GFRP spiral (mm^2^);
*D_i_*	diameter of the inner hole (mm);
*D_s_*	diameter of the GFRP spirals (center-to-center of spirals) (mm);
*D*	diameter of the whole concrete column section (mm);
*E_FRP_*	elastic modulus of the GFRP rebars (MPa);
*f*_c_^’^	compressive strength of concrete cylinder (MPa);
*f*_cc_^’^	confined concrete stress (MPa);
*f*_c_^’’^	unconfined concrete strength considering size effects (MPa);
*f_l_*	lateral confining stress (MPa);
*f_l_^’^*	effective lateral confining stress (MPa);
*f_FRP_*	ultimate tensile strength of the GFRP reinforcements (MPa);
*ε_FRP_*	ultimate tensile strain of the GFRP reinforcements (mm/mm);
*k_e_*	reduction factor regarding the vertical unconfined area between spirals (mm/mm);
*P*_*n*1_	peak load in the elastic region (kN);
*P*_*n*2_	peak load in the post-peak region (kN);
*P_f_*	load at column’s failure (kN);
s	center-to-center vertical spacing of spirals (mm);
*s^’^*	clear vertical spacing between spirals (mm);
*α*_1_	influence factor for the concrete compressive strength;
*ρ_e_*	effective longitudinal reinforcement ratio concerning the effective core area;
*L.S.R.*	hoop confinement stiffness ratio;
*ψ*	dilation angle of concrete;
*K_c_*	the shape factor of yielding surface;
*E_c_*	elastic modulus of unconfined concrete (MPa);
*σ_c_*	the axial compressive stress of concrete (MPa);
*ε_c_* *^in^*	the inelastic strain of concrete in compression (mm/mm);
*ε_c_^pl^*	the plastic strain of concrete in compression (mm/mm);
*f_t_*	maximum tensile strength of concrete (MPa);
*d_c_*	compression damage parameter;
*d_t_*	tension damage parameter;
*ε*_*c*1_	peak strain of unconfined concrete (mm/mm);
*ε*_*cc*1_	peak strain of confined concrete (mm/mm);
$d_{h}$	diameter of GFRP spiral (mm);
*ε_cu_*	ultimate strain of unconfined concrete (mm/mm);
*εc_cu_*	ultimate strain of confined concrete (mm/mm);
*f_bent_*	tensile strength of the bent GFRP bars ACI (2015) (MPa);
*ε_c1_*	axial strain of concrete (mm/mm);
*ε_oc_* ^*el*^	elastic strain of compressed unconfined concrete (mm/mm);
*ε_ot_* ^*el*^	elastic tensile strain of unconfined concrete (mm/mm);
*ε_cr_*	crack strain of unconfined concrete (mm/mm);
*ρ_v_*	ratio of GFRP spirals.
